# Supplementary material for: Integrative Proteomic Analysis Implicates Inhibition of Intracellular Protein Trafficking in Therapy-Induced Migrastasis in Prostate Cancer
Source: Proteomes. 2026 Aug 28;14(3):45. doi: 10.3390/proteomes14030045 (PMC13611321; doi:10.3390/proteomes14030045)
Supplement: Supplementary file 1 [file proteomes-14-00045-s001.zip › Extra Supplementary Files (Bioinformatics Raw Data)/Supplementary_Data_S2/Whole Cell Proteomics/Figure_2 and 3/Fig2_B/QC_PCA_PC3_72h_HQ.pdf]

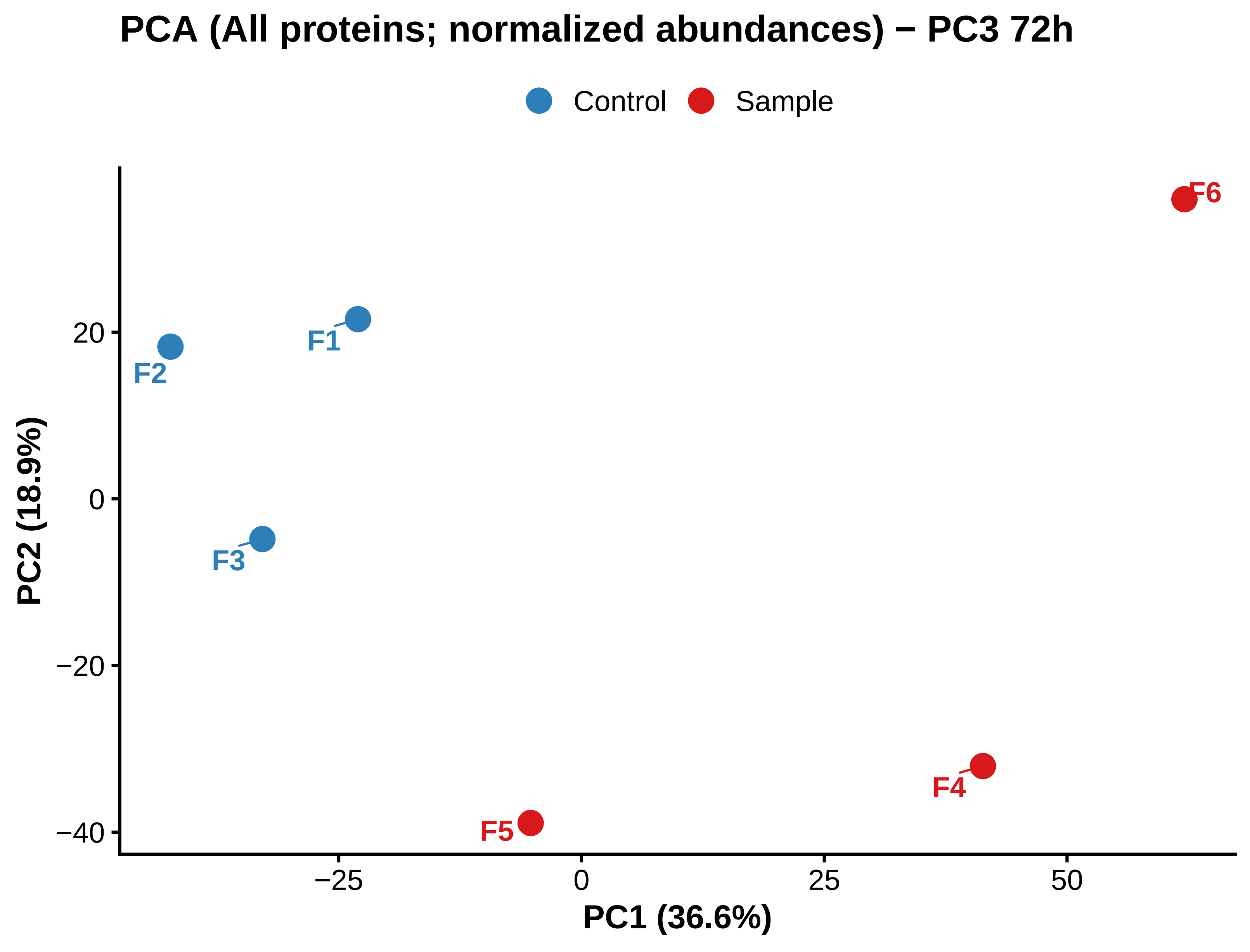

PCA (All proteins; normalized abundances) − PC3 72h
Control
Sample
F1
F2
F3
F4
F5
F6
20
0
−20
−40
−25
0
25
50
PC2 (18.9%)
PC1 (36.6%)
