# Supplementary material for: Integrative Proteomic Analysis Implicates Inhibition of Intracellular Protein Trafficking in Therapy-Induced Migrastasis in Prostate Cancer
Source: Proteomes. 2026 Aug 28;14(3):45. doi: 10.3390/proteomes14030045 (PMC13611321; doi:10.3390/proteomes14030045)
Supplement: Supplementary file 1 [file proteomes-14-00045-s001.zip › Extra Supplementary Files (Bioinformatics Raw Data)/Supplementary_Data_S2/Whole Cell Proteomics/Figure_2 and 3/Fig2_C/Volcano_PC3_72h_publication_HQ.pdf]

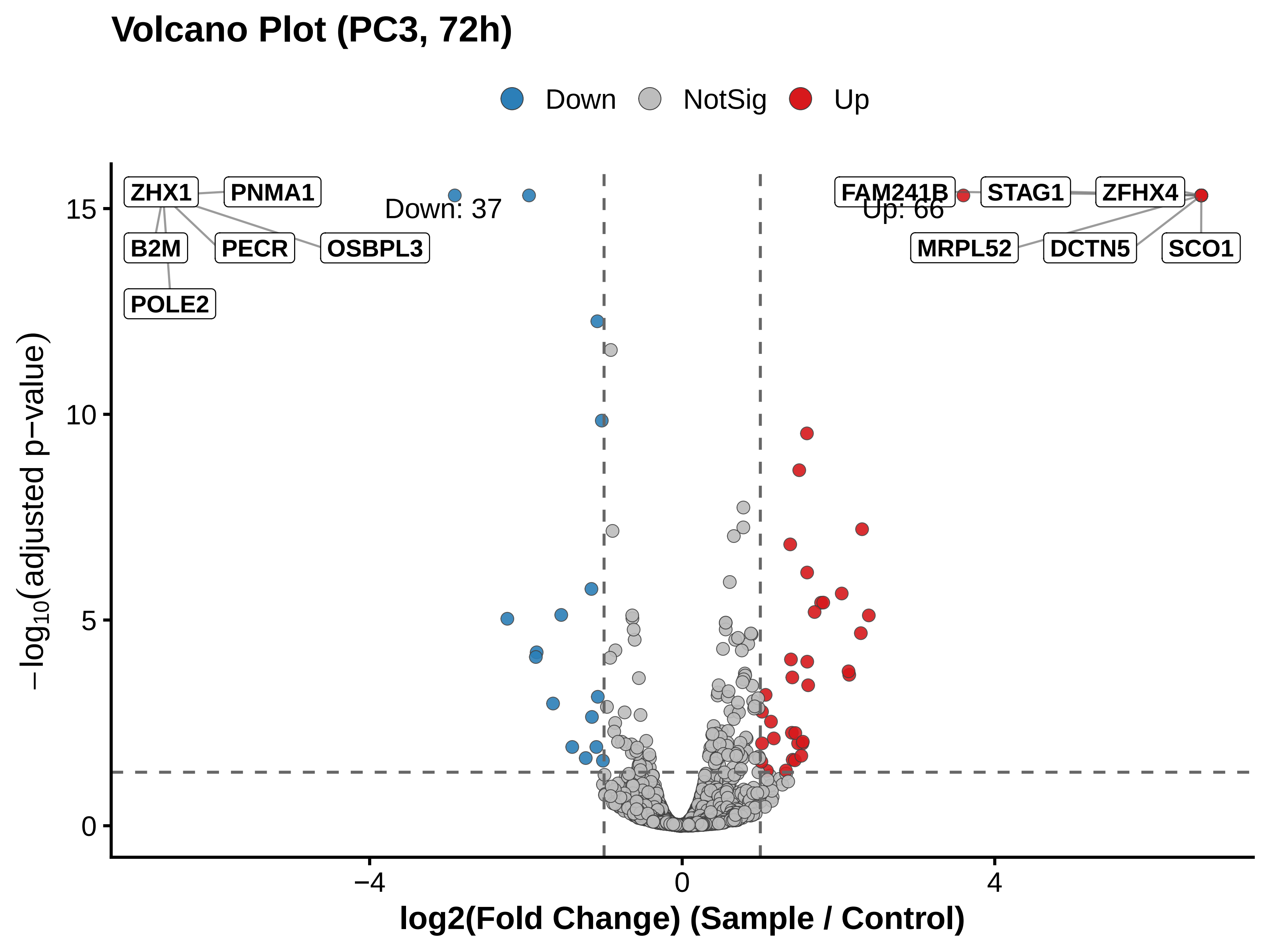

Volcano Plot (PC3, 72h)
Down
NotSig
Up
ZHX1
PNMA1
B2M
PECR
OSBPL3
POLE2
Down: 37
FAM241B
STAG1
ZFHX4
Up: 66
MRPL52
DCTN5
SCO1
15
10
5
0
−4
0
4
−log10(adjusted p−value)
log2(Fold Change) (Sample / Control)
