# Supplementary material for: Integrative Proteomic Analysis Implicates Inhibition of Intracellular Protein Trafficking in Therapy-Induced Migrastasis in Prostate Cancer
Source: Proteomes. 2026 Aug 28;14(3):45. doi: 10.3390/proteomes14030045 (PMC13611321; doi:10.3390/proteomes14030045)
Supplement: Supplementary file 1 [file proteomes-14-00045-s001.zip › Extra Supplementary Files (Bioinformatics Raw Data)/Supplementary_Data_S2/Whole Cell Proteomics/Figure_2 and 3/Fig3_A/Motility_Heatmap_log2FC_TopStable.pdf]

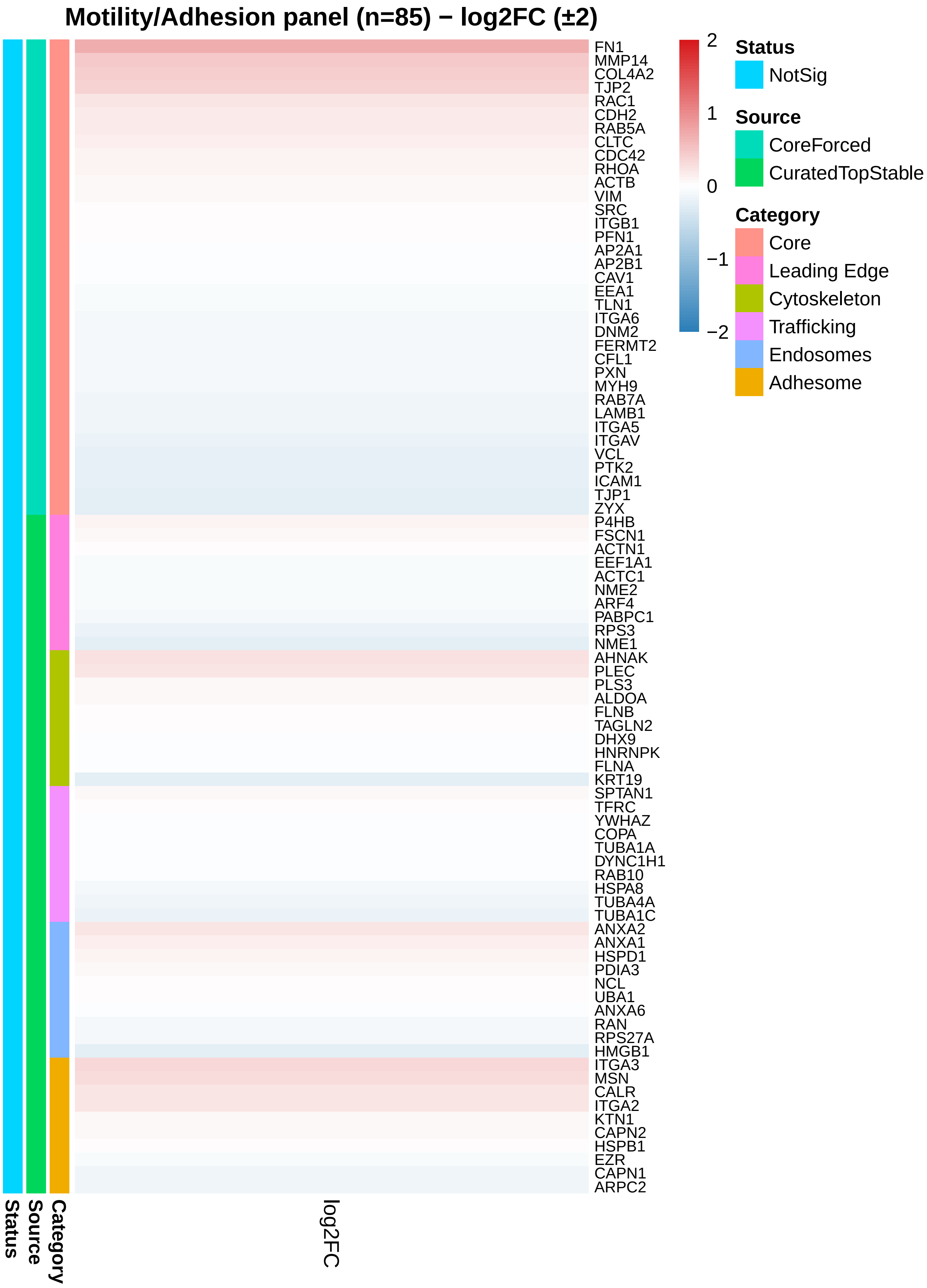
Motility/Adhesion panel (n=85) − log2FC (±2)
FN1
MMP14
COL4A2
TJP2
RAC1
CDH2
RAB5A
CLTC
CDC42
RHOA
ACTB
VIM
SRC
ITGB1
PFN1
AP2A1
AP2B1
CAV1
EEA1
TLN1
ITGA6
DNM2
FERMT2
CFL1
PXN
MYH9
RAB7A
LAMB1
ITGA5
ITGAV
VCL
PTK2
ICAM1
TJP1
ZYX
P4HB
FSCN1
ACTN1
EEF1A1
ACTC1
NME2
ARF4
PABPC1
RPS3
NME1
AHNAK
PLEC
PLS3
ALDOA
FLNB
TAGLN2
DHX9
HNRNPK
FLNA
KRT19
SPTAN1
TFRC
YWHAZ
COPA
TUBA1A
DYNC1H1
RAB10
HSPA8
TUBA4A
TUBA1C
ANXA2
ANXA1
HSPD1
PDIA3
NCL
UBA1
ANXA6
RAN
RPS27A
HMGB1
ITGA3
MSN
CALR
ITGA2
KTN1
CAPN2
HSPB1
EZR
CAPN1
ARPC2
Status
Source
Category
log2FC
2
1
0
−1
−2
Status
NotSig
Source
CoreForced
CuratedTopStable
Category
Core
Leading Edge
Cytoskeleton
Trafficking
Endosomes
Adhesome
