# Supplementary material for: Integrative Proteomic Analysis Implicates Inhibition of Intracellular Protein Trafficking in Therapy-Induced Migrastasis in Prostate Cancer
Source: Proteomes. 2026 Aug 28;14(3):45. doi: 10.3390/proteomes14030045 (PMC13611321; doi:10.3390/proteomes14030045)
Supplement: Supplementary file 1 [file proteomes-14-00045-s001.zip › Extra Supplementary Files (Bioinformatics Raw Data)/Supplementary_Data_S2/Whole Cell Proteomics/Figure_2 and 3/Fig3_B/Enrichr_results_bar_whole cell_down.png 1,360×746 pixels.pdf]

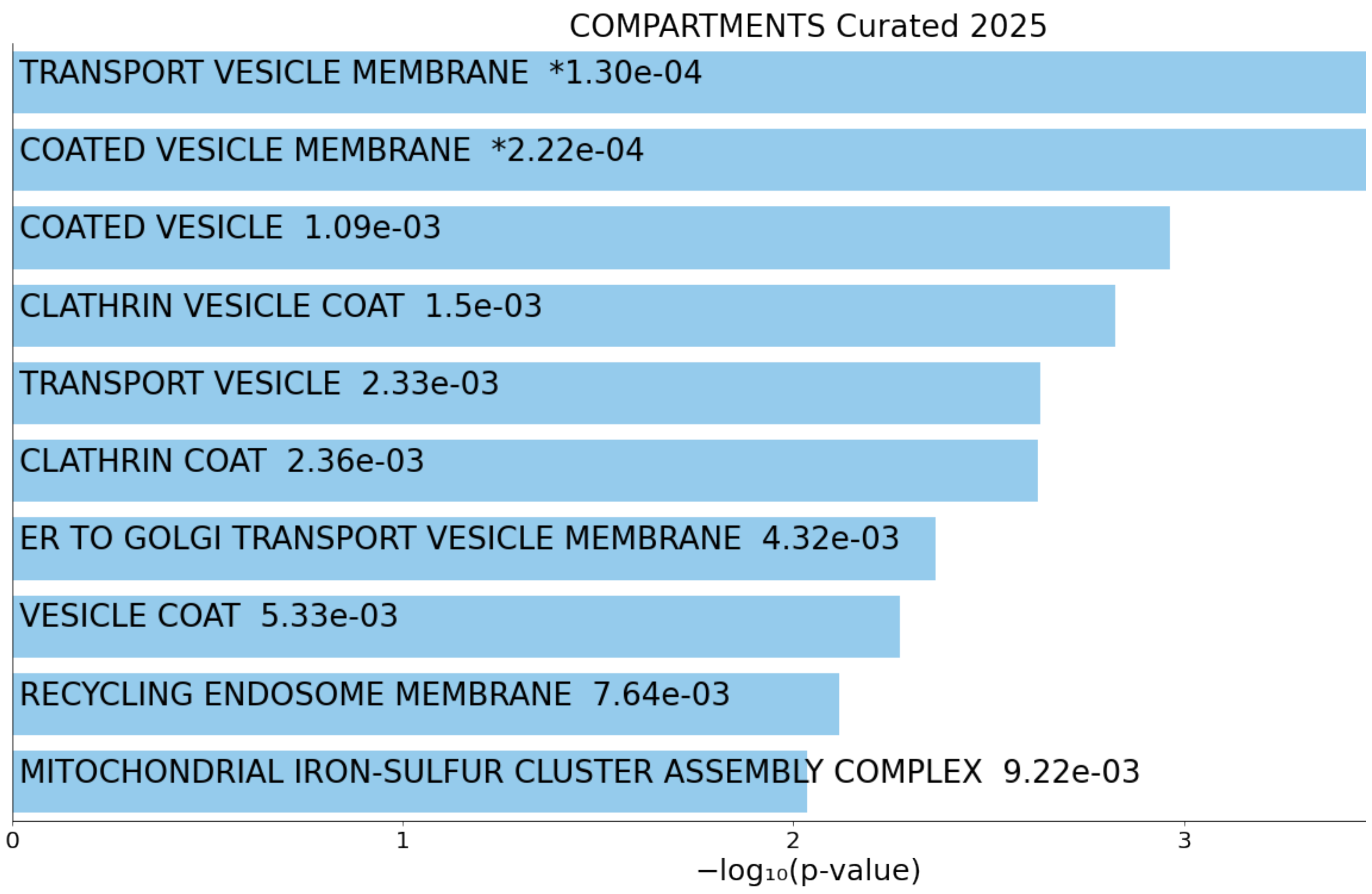
COMPARTMENTS Curated 2025
TRANSPORT VESICLE MEMBRANE *1.30e-04
COATED VESICLE MEMBRANE *2.22e-04
COATED VESICLE 1.09e-03
CLATHRIN VESICLE COAT 1.5e-03
TRANSPORT VESICLE 2.33e-03
CLATHRIN COAT 2.36e-03
ER TO GOLGI TRANSPORT VESICLE MEMBRANE 4.32e-03
VESICLE COAT 5.33e-03
RECYCLING ENDOSOME MEMBRANE 7.64e-03
MITOCHONDRIAL IRON-SULFUR CLUSTER ASSEMBLY COMPLEX 9.22e-03
0
1
2
3
−log10(p-value)
