# Supplementary material for: Integrative Proteomic Analysis Implicates Inhibition of Intracellular Protein Trafficking in Therapy-Induced Migrastasis in Prostate Cancer
Source: Proteomes. 2026 Aug 28;14(3):45. doi: 10.3390/proteomes14030045 (PMC13611321; doi:10.3390/proteomes14030045)
Supplement: Supplementary file 1 [file proteomes-14-00045-s001.zip › Extra Supplementary Files (Bioinformatics Raw Data)/Supplementary_Data_S2/Whole Cell Proteomics/Figure_2 and 3/Fig3_C/Enrichr_results_bar_whole cell_up.png 1,360×746 pixels.pdf]

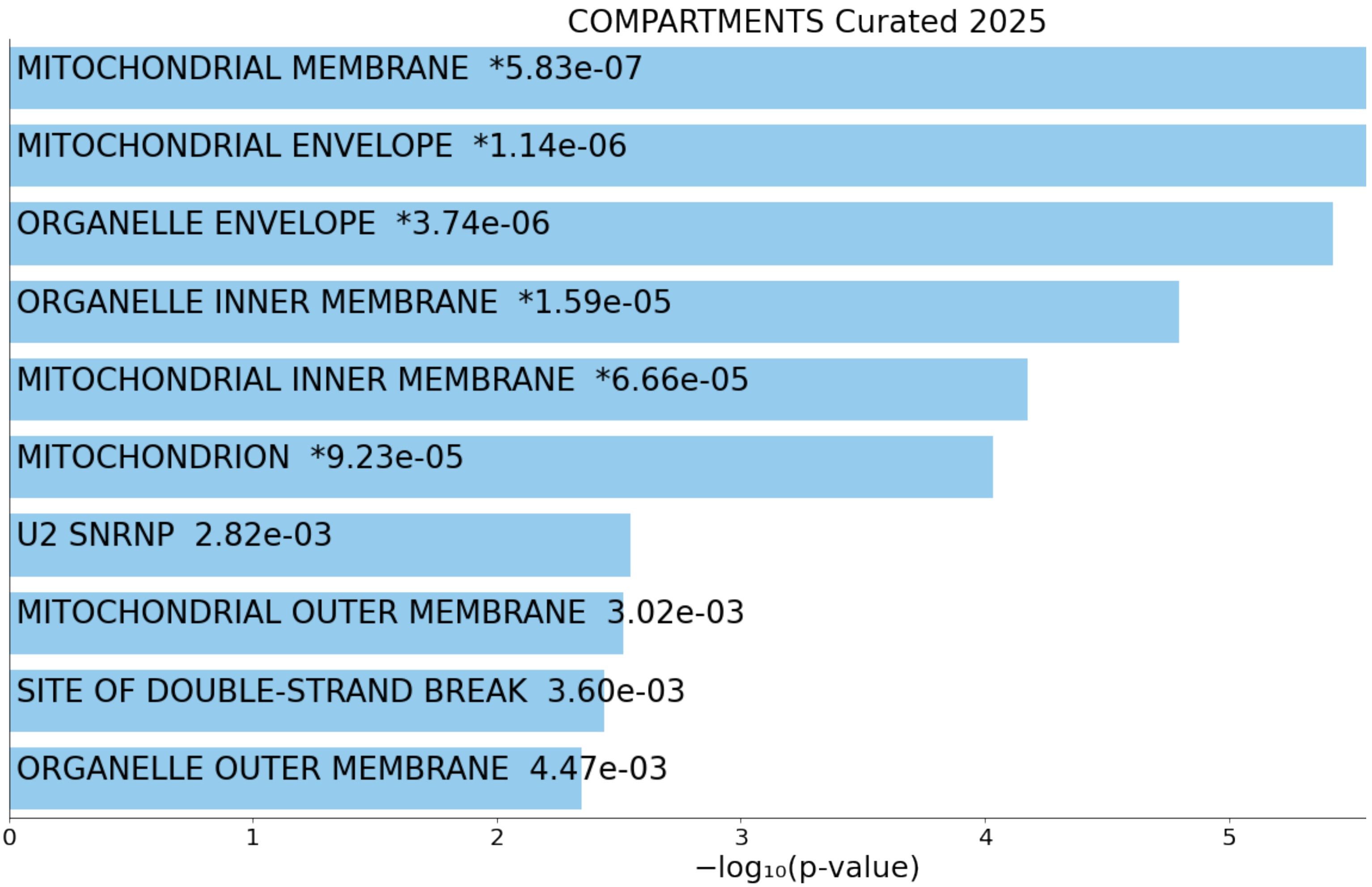
COMPARTMENTS Curated 2025
MITOCHONDRIAL MEMBRANE *5.83e-07
MITOCHONDRIAL ENVELOPE *1.14e-06
ORGANELLE ENVELOPE *3.74e-06
ORGANELLE INNER MEMBRANE *1.59e-05
MITOCHONDRIAL INNER MEMBRANE *6.66e-05
MITOCHONDRION *9.23e-05
U2 SNRNP 2.82e-03
MITOCHONDRIAL OUTER MEMBRANE 3.02e-03
SITE OF DOUBLE-STRAND BREAK 3.60e-03
ORGANELLE OUTER MEMBRANE 4.47e-03
0
1
2
3
4
5
−log10(p-value)
