# Supplementary material for: Integrative Proteomic Analysis Implicates Inhibition of Intracellular Protein Trafficking in Therapy-Induced Migrastasis in Prostate Cancer
Source: Proteomes. 2026 Aug 28;14(3):45. doi: 10.3390/proteomes14030045 (PMC13611321; doi:10.3390/proteomes14030045)
Supplement: Supplementary file 1 [file proteomes-14-00045-s001.zip › Extra Supplementary Files (Bioinformatics Raw Data)/Supplementary_Data_S3/Membrane proteomics/Figure_4 and 5/Fig4_B/PCA_PC1_vs_PC2_HQ.pdf]

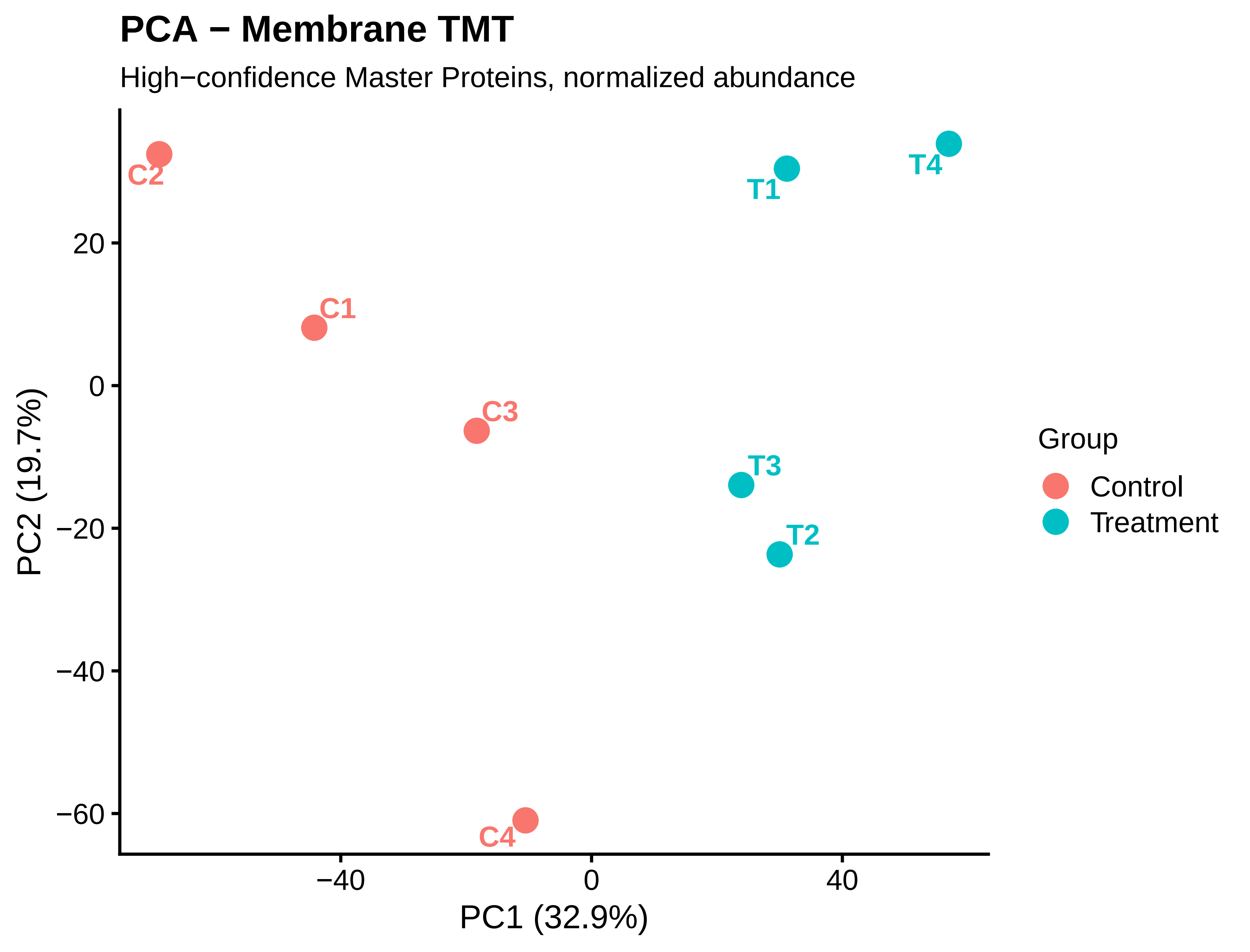
PCA − Membrane TMT
High−confidence Master Proteins, normalized abundance
PC2 (19.7%)
PC1 (32.9%)
20
0
−20
−40
−60
−40
0
40
C1
C2
C3
C4
T1
T2
T3
T4
Group
Control
Treatment
