# Supplementary material for: Integrative Proteomic Analysis Implicates Inhibition of Intracellular Protein Trafficking in Therapy-Induced Migrastasis in Prostate Cancer
Source: Proteomes. 2026 Aug 28;14(3):45. doi: 10.3390/proteomes14030045 (PMC13611321; doi:10.3390/proteomes14030045)
Supplement: Supplementary file 1 [file proteomes-14-00045-s001.zip › Extra Supplementary Files (Bioinformatics Raw Data)/Supplementary_Data_S3/Membrane proteomics/Figure_4 and 5/Fig4_C/QC_GO_CC_top100_dotplot_HQ.pdf]

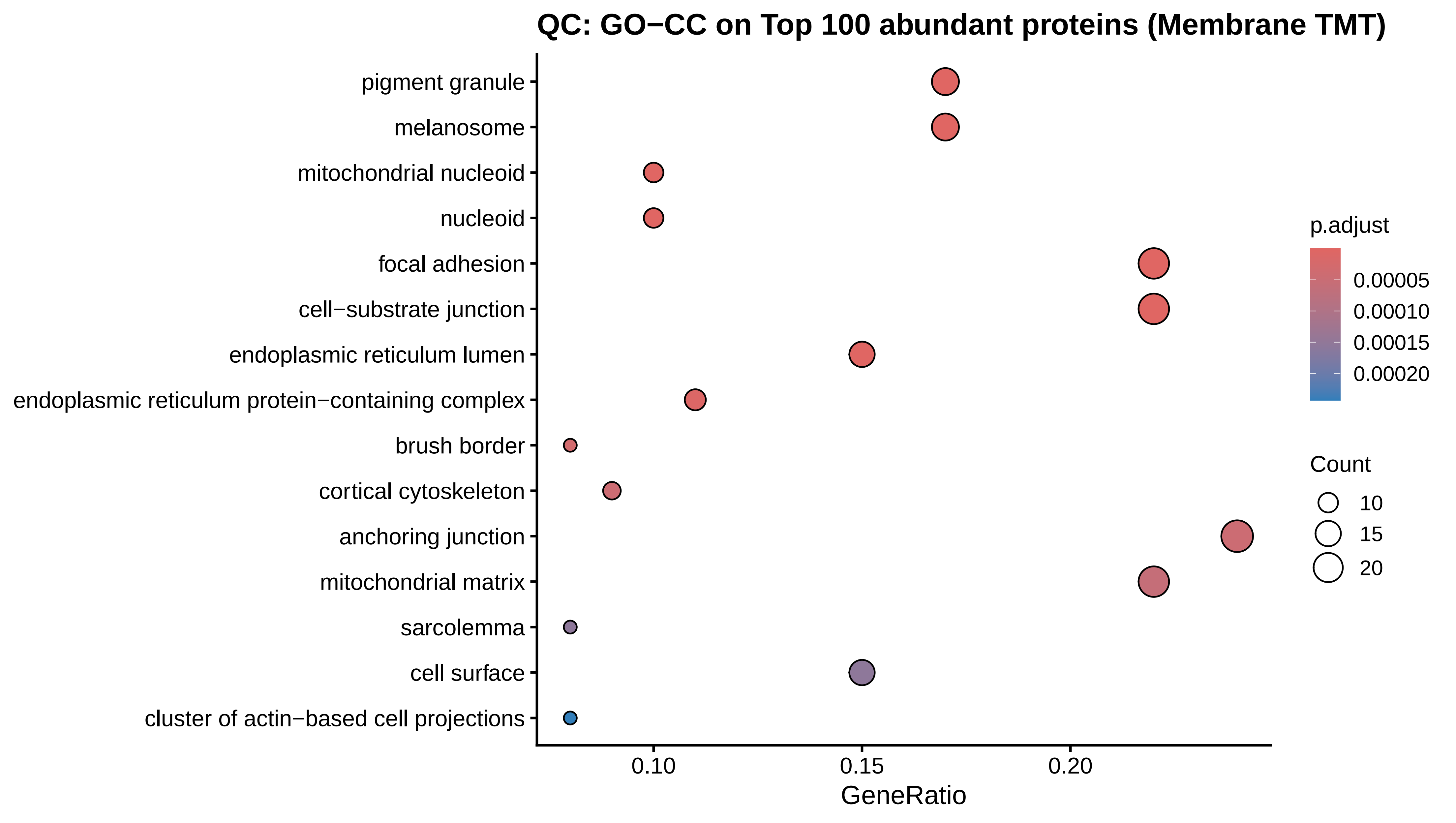
QC: GO−CC on Top 100 abundant proteins (Membrane TMT)
pigment granule
melanosome
mitochondrial nucleoid
nucleoid
focal adhesion
cell−substrate junction
endoplasmic reticulum lumen
endoplasmic reticulum protein−containing complex
brush border
cortical cytoskeleton
anchoring junction
mitochondrial matrix
sarcolemma
cell surface
cluster of actin−based cell projections
0.10
0.15
0.20
GeneRatio
p.adjust
0.00005
0.00010
0.00015
0.00020
Count
10
15
20
