# Supplementary material for: Integrative Proteomic Analysis Implicates Inhibition of Intracellular Protein Trafficking in Therapy-Induced Migrastasis in Prostate Cancer
Source: Proteomes. 2026 Aug 28;14(3):45. doi: 10.3390/proteomes14030045 (PMC13611321; doi:10.3390/proteomes14030045)
Supplement: Supplementary file 1 [file proteomes-14-00045-s001.zip › Extra Supplementary Files (Bioinformatics Raw Data)/Supplementary_Data_S3/Membrane proteomics/Figure_4 and 5/Fig5_B/Volcano_MembraneTMT_HighStringency.pdf]

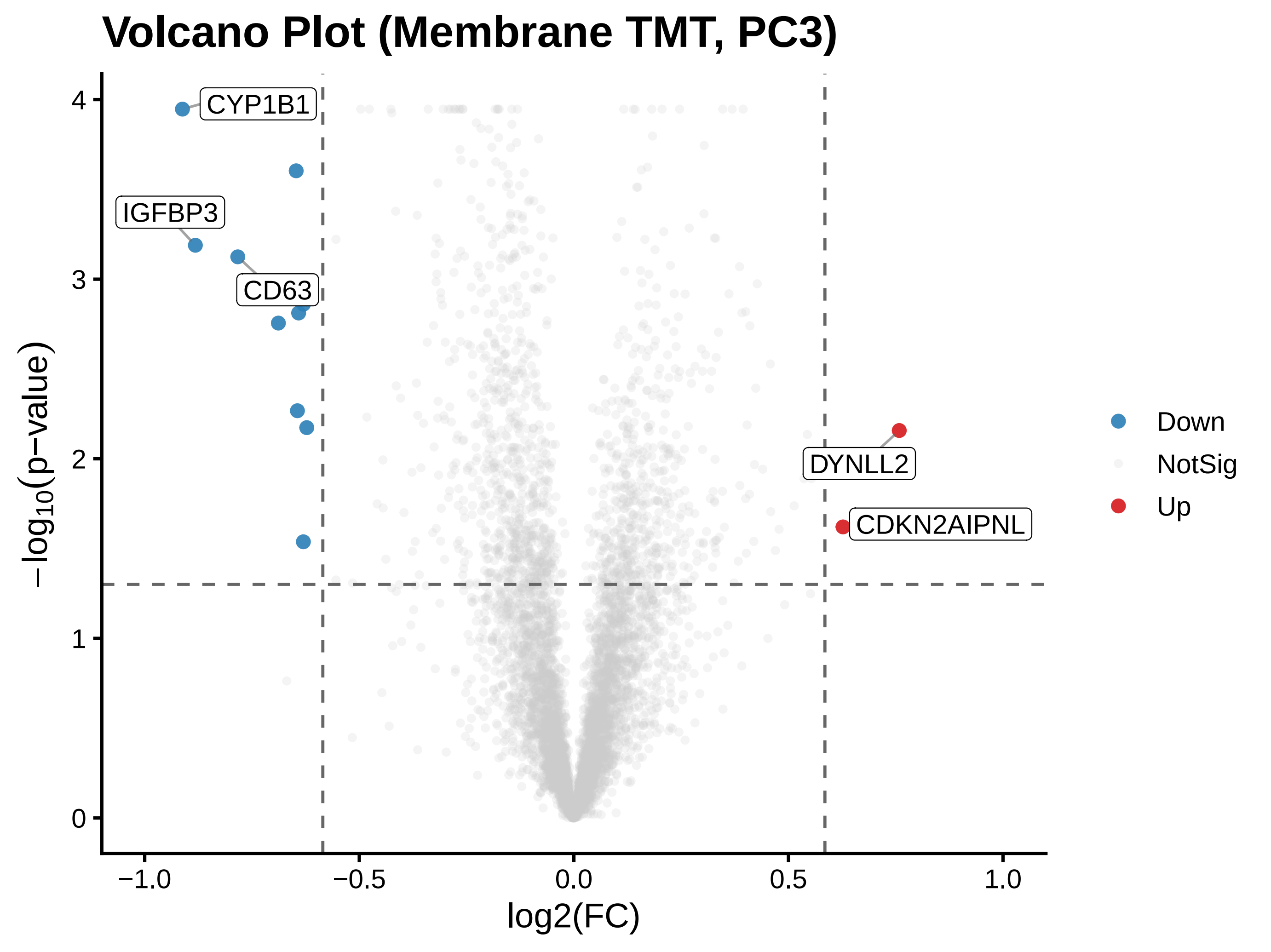

Volcano Plot (Membrane TMT, PC3)
CYP1B1
IGFBP3
CD63
DYNLL2
CDKN2AIPNL
−log10(p−value)
4
3
2
1
0
−1.0
−0.5
0.0
0.5
1.0
log2(FC)
Down
NotSig
Up
