# Supplementary material for: Integrative Proteomic Analysis Implicates Inhibition of Intracellular Protein Trafficking in Therapy-Induced Migrastasis in Prostate Cancer
Source: Proteomes. 2026 Aug 28;14(3):45. doi: 10.3390/proteomes14030045 (PMC13611321; doi:10.3390/proteomes14030045)
Supplement: Supplementary file 1 [file proteomes-14-00045-s001.zip › Extra Supplementary Files (Bioinformatics Raw Data)/Supplementary_Data_S3/Membrane proteomics/Figure_4 and 5/Fig5_C/1a_std_up_BP_dotplot.pdf]

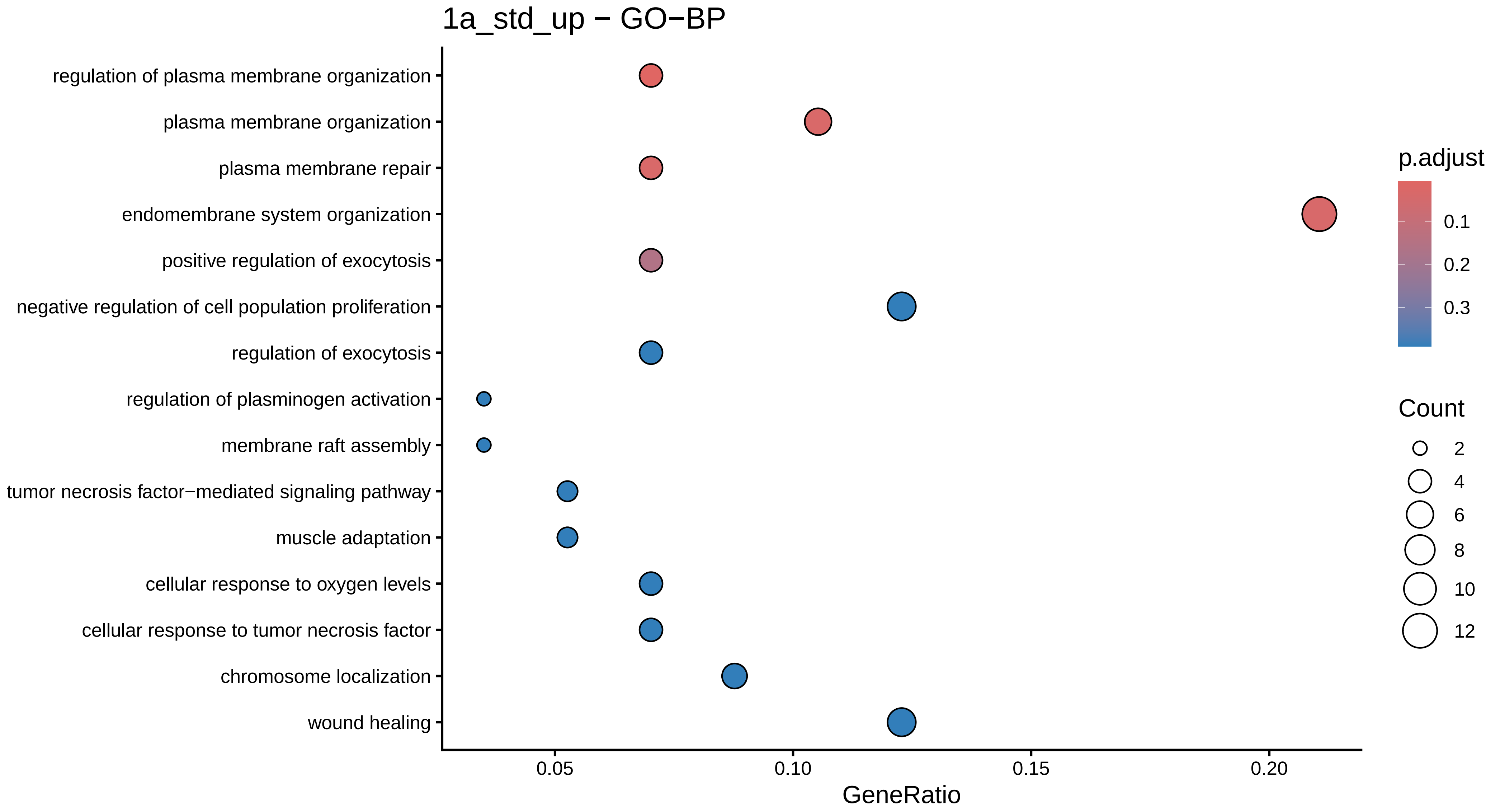
1a_std_up − GO−BP
regulation of plasma membrane organization
plasma membrane organization
plasma membrane repair
endomembrane system organization
positive regulation of exocytosis
negative regulation of cell population proliferation
regulation of exocytosis
regulation of plasminogen activation
membrane raft assembly
tumor necrosis factor−mediated signaling pathway
muscle adaptation
cellular response to oxygen levels
cellular response to tumor necrosis factor
chromosome localization
wound healing
0.05
0.10
0.15
0.20
GeneRatio
p.adjust
0.1
0.2
0.3
Count
2
4
6
8
10
12
