# Supplementary material for: Integrative Proteomic Analysis Implicates Inhibition of Intracellular Protein Trafficking in Therapy-Induced Migrastasis in Prostate Cancer
Source: Proteomes. 2026 Aug 28;14(3):45. doi: 10.3390/proteomes14030045 (PMC13611321; doi:10.3390/proteomes14030045)
Supplement: Supplementary file 1 [file proteomes-14-00045-s001.zip › Extra Supplementary Files (Bioinformatics Raw Data)/Supplementary_Data_S3/Membrane proteomics/Figure_4 and 5/Fig5_D/1b_std_down_BP_dotplot.pdf]

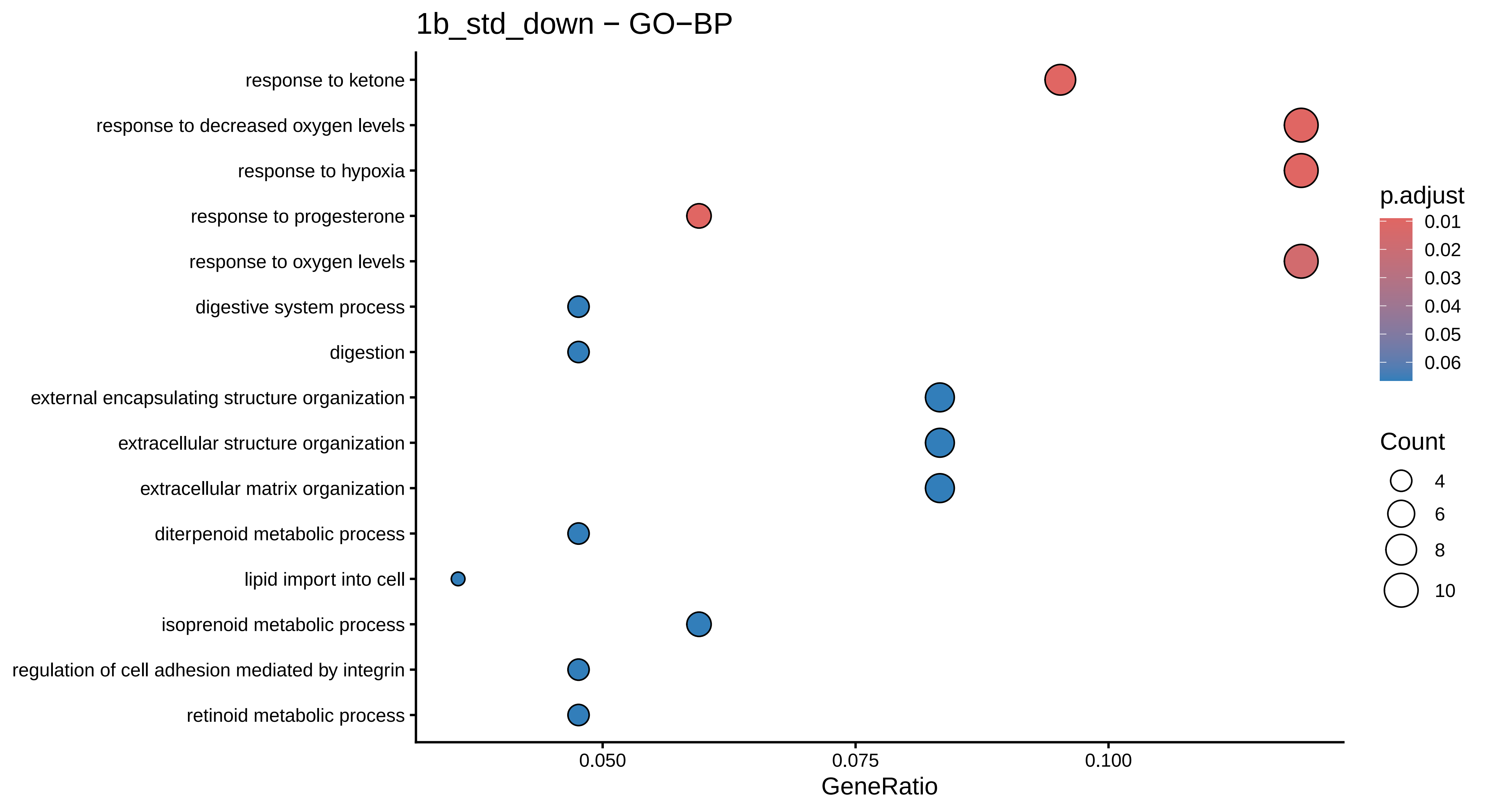

1b_std_down − GO−BP
response to ketone
response to decreased oxygen levels
response to hypoxia
response to progesterone
response to oxygen levels
digestive system process
digestion
external encapsulating structure organization
extracellular structure organization
extracellular matrix organization
diterpenoid metabolic process
lipid import into cell
isoprenoid metabolic process
regulation of cell adhesion mediated by integrin
retinoid metabolic process
0.050
0.075
0.100
GeneRatio
p.adjust
0.01
0.02
0.03
0.04
0.05
0.06
Count
4
6
8
10
