# Supplementary material for: Integrative Proteomic Analysis Implicates Inhibition of Intracellular Protein Trafficking in Therapy-Induced Migrastasis in Prostate Cancer
Source: Proteomes. 2026 Aug 28;14(3):45. doi: 10.3390/proteomes14030045 (PMC13611321; doi:10.3390/proteomes14030045)
Supplement: Supplementary file 1 [file proteomes-14-00045-s001.zip › Extra Supplementary Files (Bioinformatics Raw Data)/Supplementary_Data_S4/Time-course Phosphoproteomics/Figure_7 and 8/Fig7_A/PCA_PC1_vs_PC2.pdf]

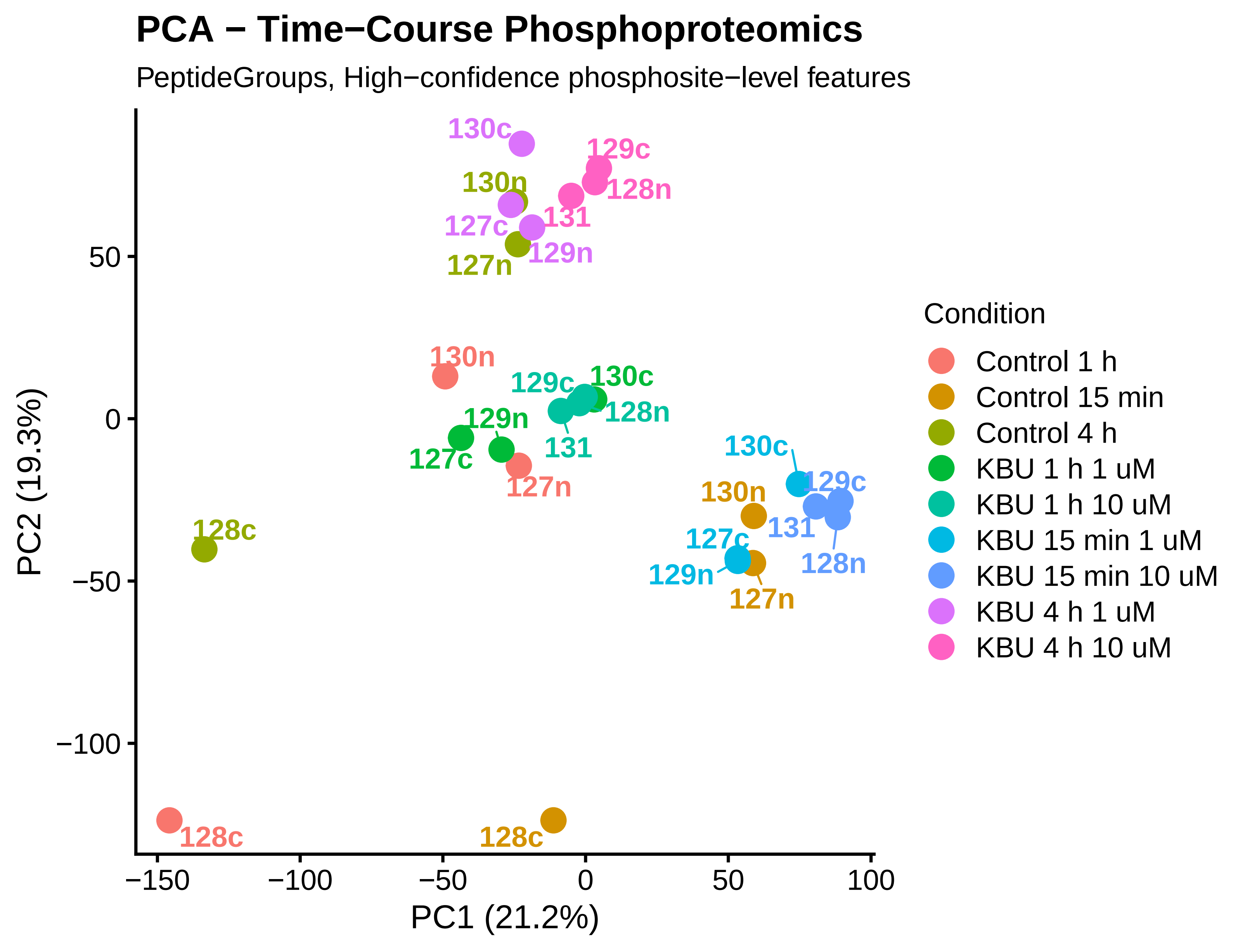
PCA − Time−Course Phosphoproteomics
PeptideGroups, High−confidence phosphosite−level features
PC2 (19.3%)
PC1 (21.2%)
50
0
−50
−100
−150
−100
−50
0
50
100
130c
129c
130n
128n
131
127c
129n
127n
130n
129c
130c
129n
128n
131
127c
127n
130c
129c
130n
131
127c
128n
129n
127n
128c
128c
128c
Condition
Control 1 h
Control 15 min
Control 4 h
KBU 1 h 1 uM
KBU 1 h 10 uM
KBU 15 min 1 uM
KBU 15 min 10 uM
KBU 4 h 1 uM
KBU 4 h 10 uM
