# Supplementary material for: Integrative Proteomic Analysis Implicates Inhibition of Intracellular Protein Trafficking in Therapy-Induced Migrastasis in Prostate Cancer
Source: Proteomes. 2026 Aug 28;14(3):45. doi: 10.3390/proteomes14030045 (PMC13611321; doi:10.3390/proteomes14030045)
Supplement: Supplementary file 1 [file proteomes-14-00045-s001.zip › Extra Supplementary Files (Bioinformatics Raw Data)/Supplementary_Data_S4/Time-course Phosphoproteomics/Figure_7 and 8/Fig7_B/Fig1_ResidueDistribution_Main_UniqueSites_Bar.pdf]

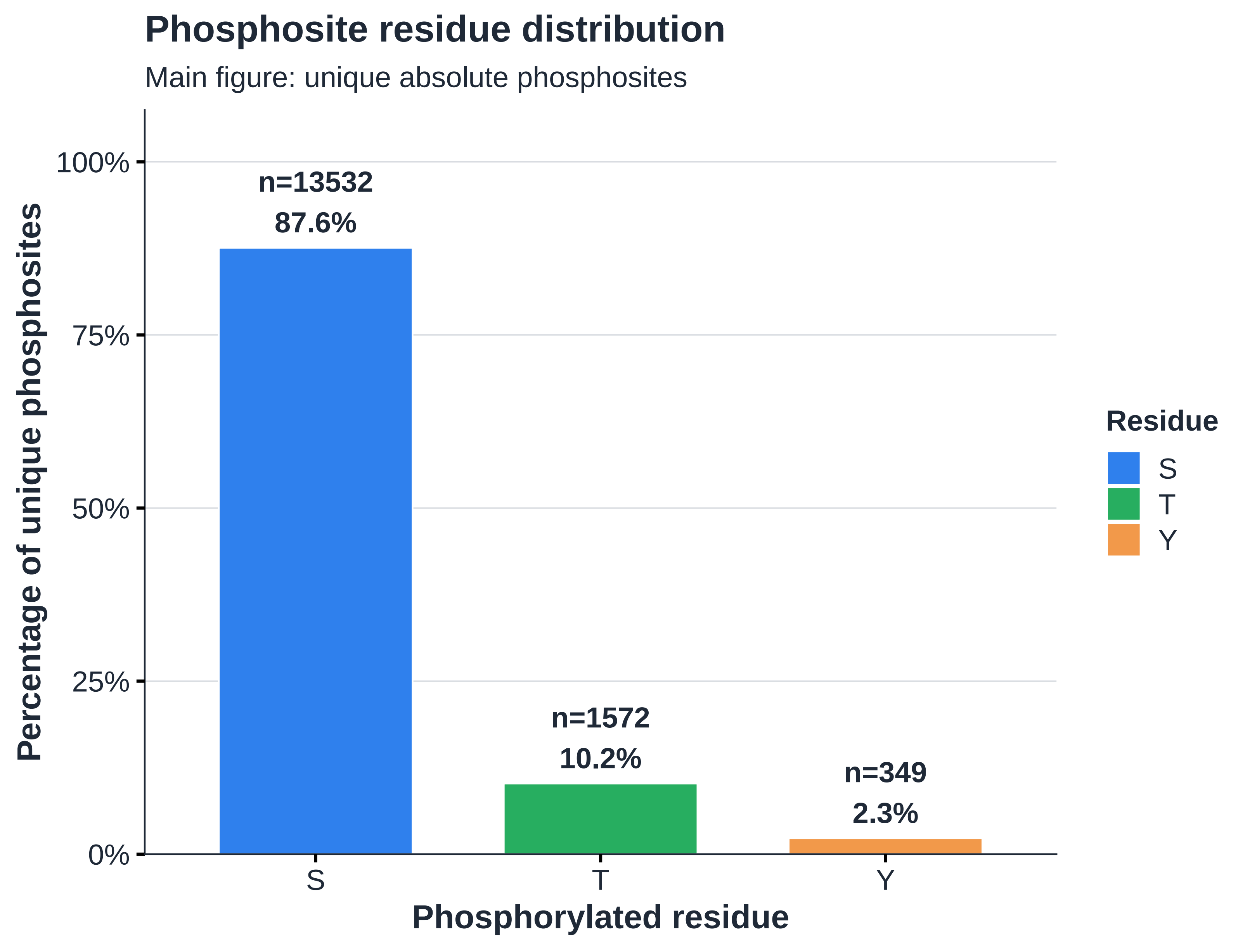
Phosphosite residue distribution
Main figure: unique absolute phosphosites
Percentage of unique phosphosites
100%
75%
50%
25%
0%
n=13532
87.6%
n=1572
10.2%
n=349
2.3%
S
T
Y
Phosphorylated residue
Residue
S
T
Y
