# Supplementary material for: Integrative Proteomic Analysis Implicates Inhibition of Intracellular Protein Trafficking in Therapy-Induced Migrastasis in Prostate Cancer
Source: Proteomes. 2026 Aug 28;14(3):45. doi: 10.3390/proteomes14030045 (PMC13611321; doi:10.3390/proteomes14030045)
Supplement: Supplementary file 1 [file proteomes-14-00045-s001.zip › Extra Supplementary Files (Bioinformatics Raw Data)/Supplementary_Data_S4/Time-course Phosphoproteomics/Figure_7 and 8/Fig7_C/Fig4_Venn_UniqueSignificantPhosphosites_10uM_vsCtrl.pdf]

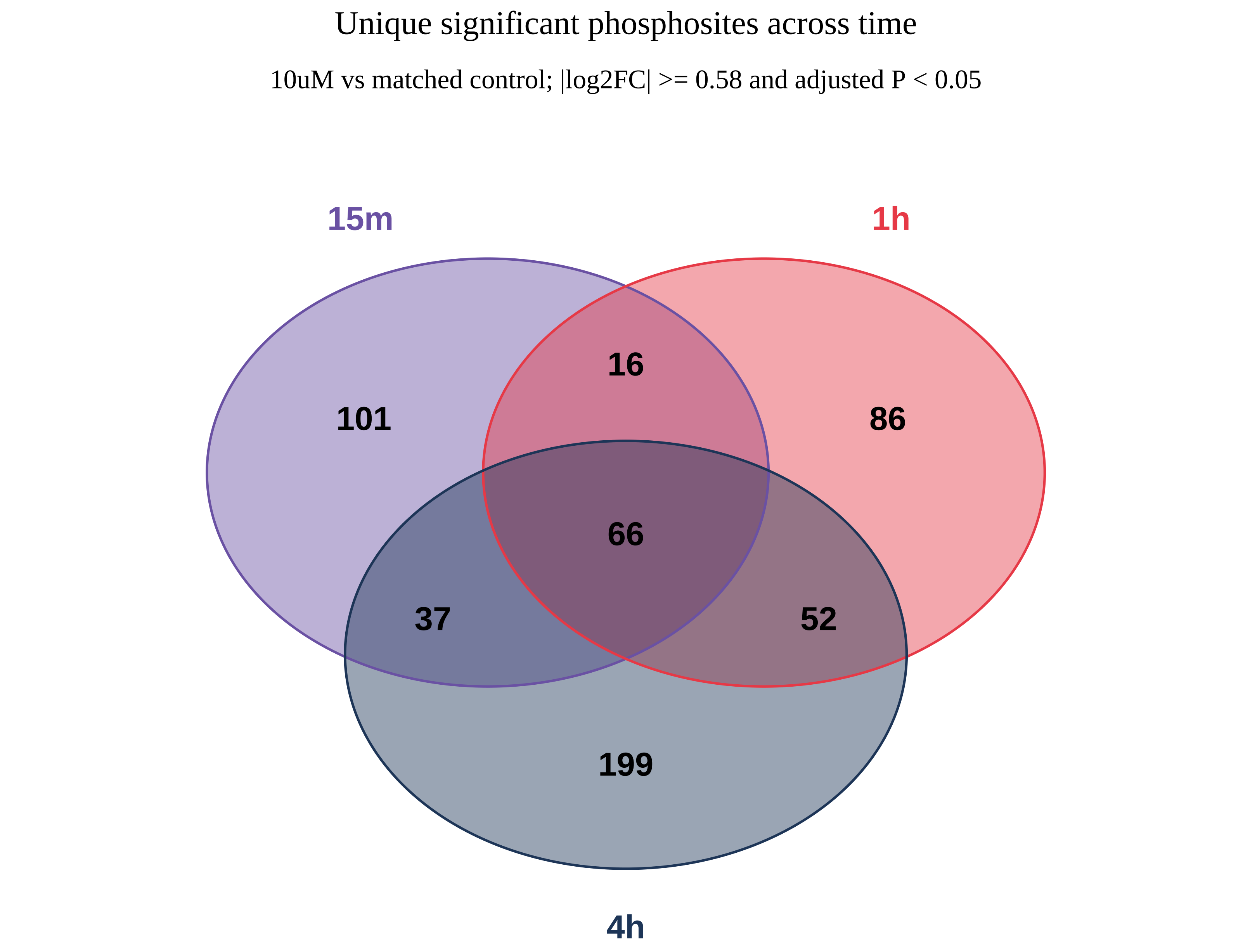

Unique significant phosphosites across time
10uM vs matched control; |log2FC| >= 0.58 and adjusted P < 0.05
15m
1h
4h
101
16
86
66
37
52
199
