# Supplementary material for: Integrative Proteomic Analysis Implicates Inhibition of Intracellular Protein Trafficking in Therapy-Induced Migrastasis in Prostate Cancer
Source: Proteomes. 2026 Aug 28;14(3):45. doi: 10.3390/proteomes14030045 (PMC13611321; doi:10.3390/proteomes14030045)
Supplement: Supplementary file 1 [file proteomes-14-00045-s001.zip › Extra Supplementary Files (Bioinformatics Raw Data)/Supplementary_Data_S4/Time-course Phosphoproteomics/Figure_7 and 8/Fig7_D/Fig3_ResidueDistribution_Significant_UniqueSites_byTime.pdf]

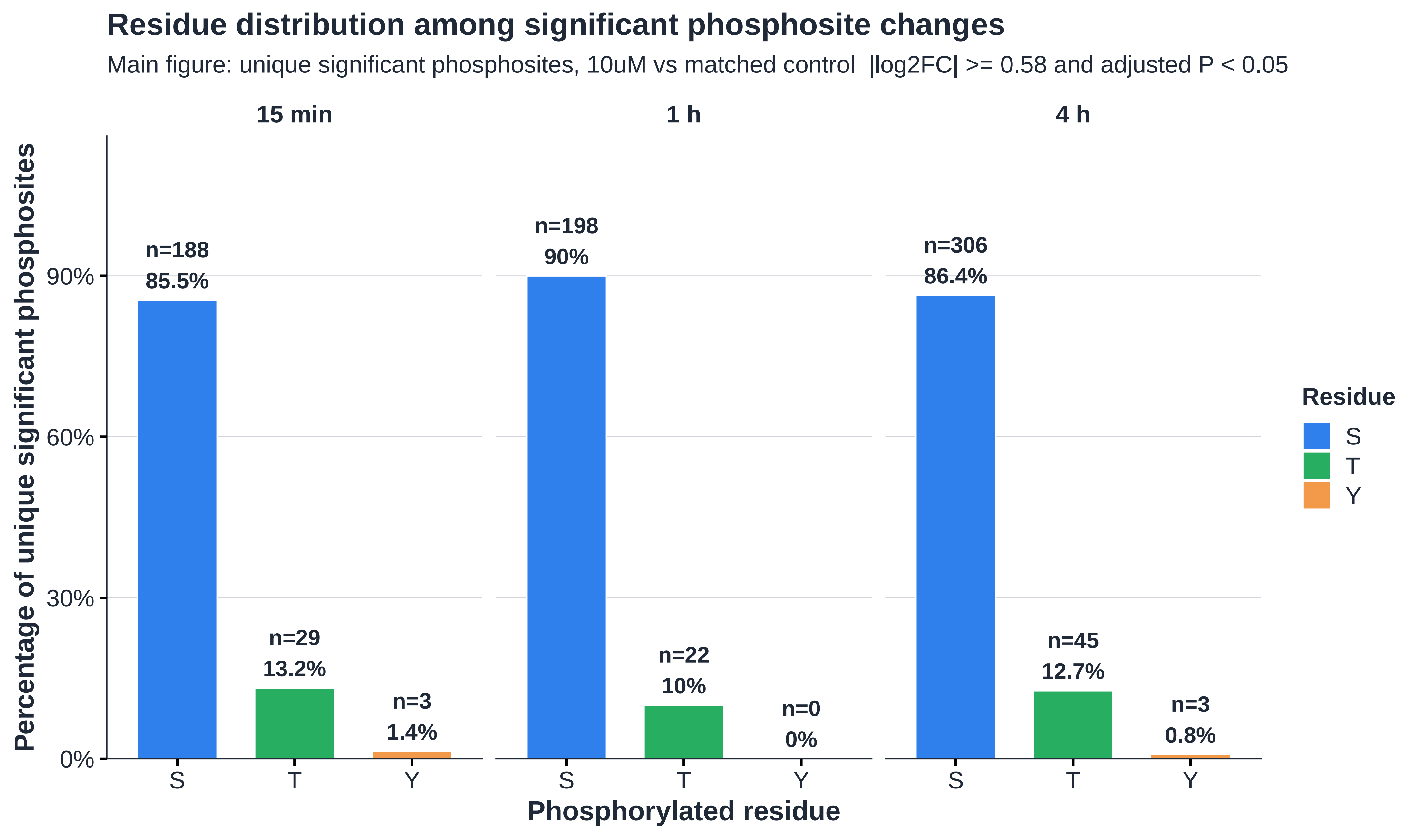
Residue distribution among significant phosphosite changes
Main figure: unique significant phosphosites, 10uM vs matched control |log2FC| >= 0.58 and adjusted P < 0.05
15 min
1 h
4 h
Percentage of unique significant phosphosites
90%
60%
30%
0%
n=188
85.5%
n=29
13.2%
n=3
1.4%
n=198
90%
n=22
10%
n=0
0%
n=306
86.4%
n=45
12.7%
n=3
0.8%
S
T
Y
Phosphorylated residue
Residue
S
T
Y
