# Supplementary material for: Integrative Proteomic Analysis Implicates Inhibition of Intracellular Protein Trafficking in Therapy-Induced Migrastasis in Prostate Cancer
Source: Proteomes. 2026 Aug 28;14(3):45. doi: 10.3390/proteomes14030045 (PMC13611321; doi:10.3390/proteomes14030045)
Supplement: Supplementary file 1 [file proteomes-14-00045-s001.zip › Extra Supplementary Files (Bioinformatics Raw Data)/Supplementary_Data_S4/Time-course Phosphoproteomics/Figure_9/Fig9_B/KSEA_MAPK14_p38_Activity_4h.pdf]

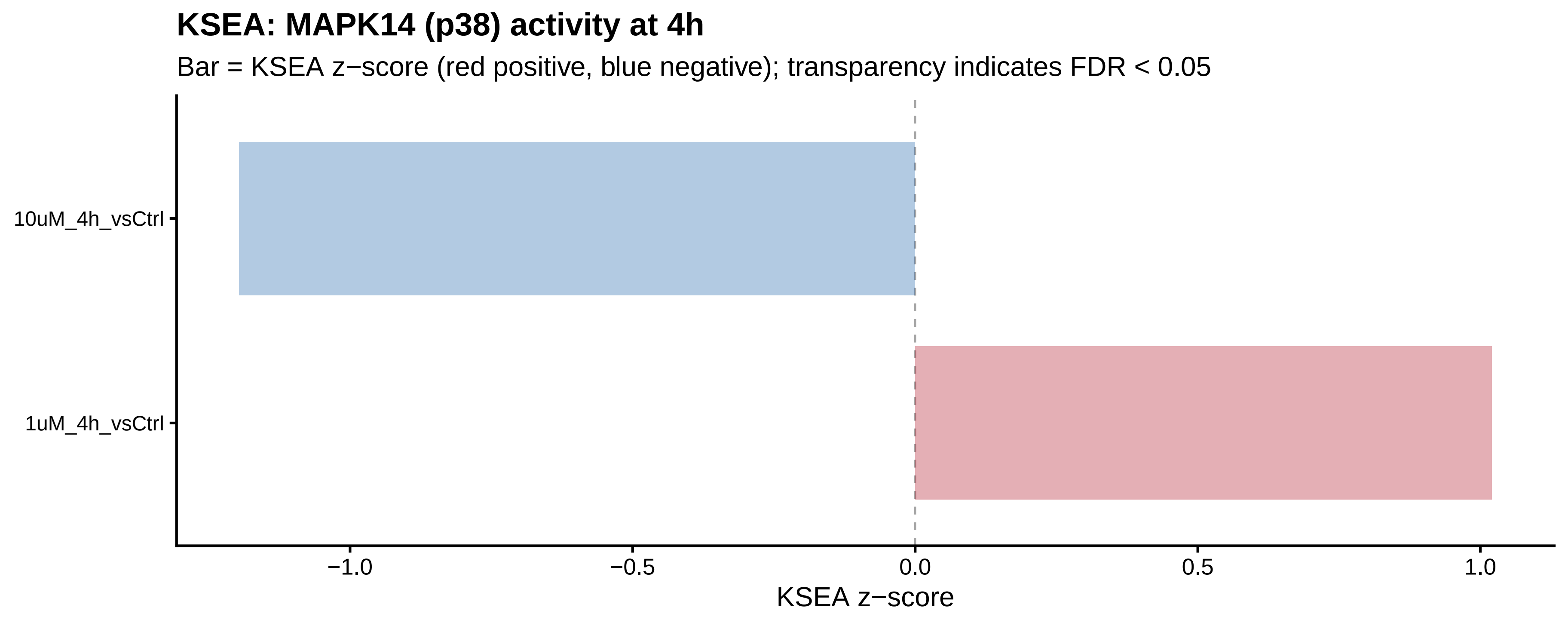
KSEA: MAPK14 (p38) activity at 4h
Bar = KSEA z−score (red positive, blue negative); transparency indicates FDR < 0.05
10uM_4h_vsCtrl
1uM_4h_vsCtrl
−1.0
−0.5
0.0
0.5
1.0
KSEA z−score
