# Supplementary material for: Integrative Proteomic Analysis Implicates Inhibition of Intracellular Protein Trafficking in Therapy-Induced Migrastasis in Prostate Cancer
Source: Proteomes. 2026 Aug 28;14(3):45. doi: 10.3390/proteomes14030045 (PMC13611321; doi:10.3390/proteomes14030045)
Supplement: Supplementary file 1 [file proteomes-14-00045-s001.zip › Extra Supplementary Files (Bioinformatics Raw Data)/Supplementary_Data_S4/Time-course Phosphoproteomics/Figure_9/Fig9_C/MAPK14_p38_Sites_4h_Dumbbell.pdf]

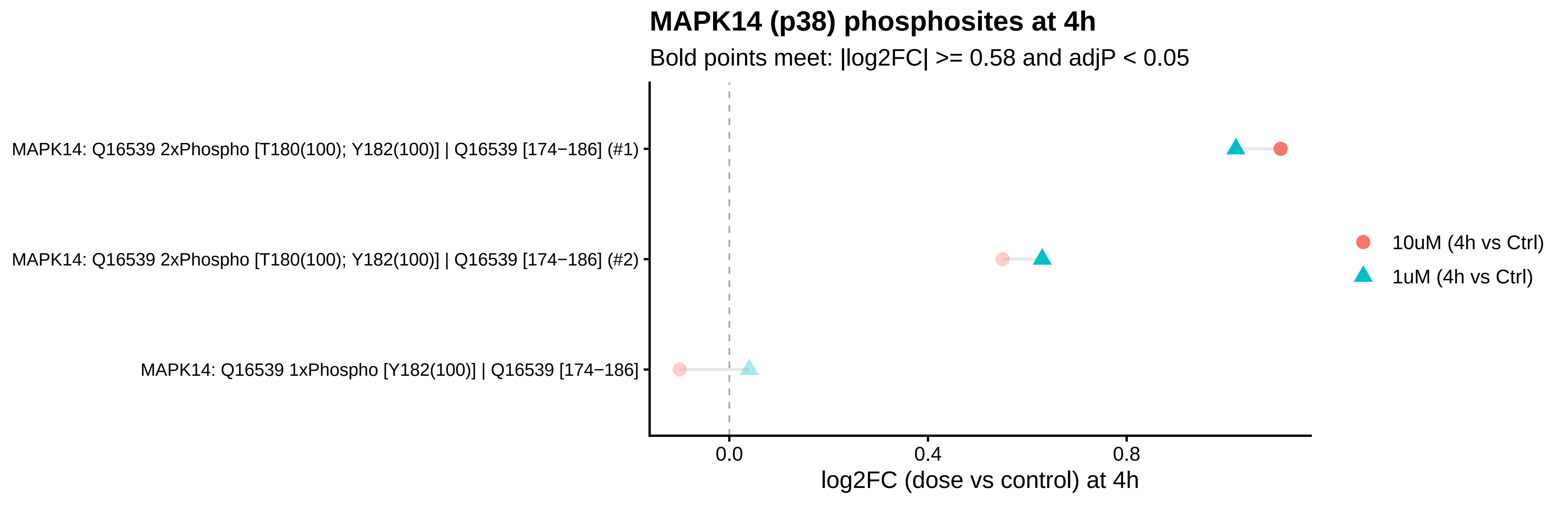
MAPK14 (p38) phosphosites at 4h
Bold points meet: |log2FC| >= 0.58 and adjP < 0.05
MAPK14: Q16539 2xPhospho [T180(100); Y182(100)] | Q16539 [174−186] (#1)
MAPK14: Q16539 2xPhospho [T180(100); Y182(100)] | Q16539 [174−186] (#2)
MAPK14: Q16539 1xPhospho [Y182(100)] | Q16539 [174−186]
0.0
0.4
0.8
log2FC (dose vs control) at 4h
10uM (4h vs Ctrl)
1uM (4h vs Ctrl)
