# Supplementary material for: Integrative Proteomic Analysis Implicates Inhibition of Intracellular Protein Trafficking in Therapy-Induced Migrastasis in Prostate Cancer
Source: Proteomes. 2026 Aug 28;14(3):45. doi: 10.3390/proteomes14030045 (PMC13611321; doi:10.3390/proteomes14030045)
Supplement: Supplementary file 1 [file proteomes-14-00045-s001.zip › Extra Supplementary Files (Bioinformatics Raw Data)/Supplementary_Data_S4/Time-course Phosphoproteomics/Figure_9/Fig9_D/Figure5D_MAPK14_Substrates_Heatmap_4h.pdf]

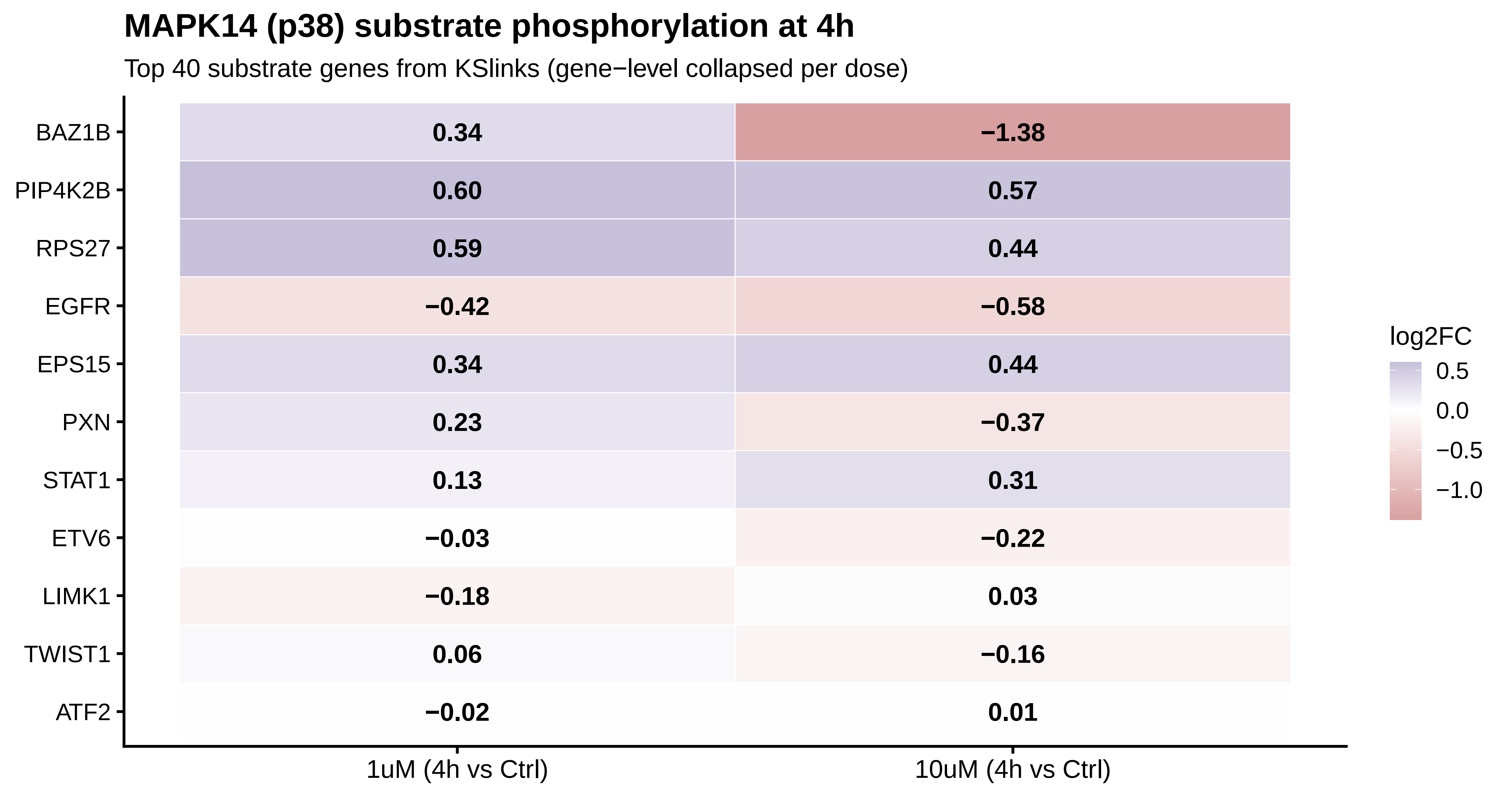
MAPK14 (p38) substrate phosphorylation at 4h
Top 40 substrate genes from KSlinks (gene−level collapsed per dose)
BAZ1B
PIP4K2B
RPS27
EGFR
EPS15
PXN
STAT1
ETV6
LIMK1
TWIST1
ATF2
0.34
−1.38
0.60
0.57
0.59
0.44
−0.42
−0.58
0.34
0.44
0.23
−0.37
0.13
0.31
−0.03
−0.22
−0.18
0.03
0.06
−0.16
−0.02
0.01
1uM (4h vs Ctrl)
10uM (4h vs Ctrl)
log2FC
0.5
0.0
−0.5
−1.0
