# Supplementary material for: Integrative Proteomic Analysis Implicates Inhibition of Intracellular Protein Trafficking in Therapy-Induced Migrastasis in Prostate Cancer
Source: Proteomes. 2026 Aug 28;14(3):45. doi: 10.3390/proteomes14030045 (PMC13611321; doi:10.3390/proteomes14030045)
Supplement: Supplementary file 1 [file proteomes-14-00045-s001.zip › Proteomics experiments Excel Sheets and LC_MS details_/Membrane Specific TMT/MSmethod_Bergan.pdf]

Orbitrap Exploris 480 Method Summary

Creator: F24NB93\Thermo Last Modified: 06/28/2022 15:35:30 by F24NB93\Thermo

Global Settings

- Use Static Source Gasses
- Use Ion Source Settings from Tune = Not Checked
- Method Duration (min)= 155
- Ion Source Type = NSI
- Spray Voltage = Static
- Spray Voltage: Positive Ion (V) = 1800
- Spray Voltage: Negative Ion (V) = 600
- Gas Mode = Static
- Infusion Mode (LC)= False
- Ion Transfer Tube Temp (°C) = 280
- APPI Lamp = Not in use
- Total Carrier Gas Flow = 4
- FAIMS Mode = Standard Resolution
- Application Mode = Peptide
- Pressure Mode = Standard
- Expected Peak Width (s) = 30
- Default Charge State = 2
- Advanced Peak Determination = False
- Xcalibur AcquireX enabled for method modifications = False

Experiment 1

- Experiment Name = MS
- Start Time (min) = 0
- End Time (min) = 155
- Cycle Time (sec) = 2

Scan MasterScan

- Orbitrap Resolution = 120000
- Scan Range (m/z) = 350-1200
- Microscans = 1
- AGC Target = Standard
- RF Lens(%) = 40
- Maximum Injection Time Mode = Auto
- FAIMS Voltages On = True
- DataType = Profile
- FAIMS CV = -45
- Polarity = Positive
- Source Fragmentation = False
- Scan Description =

Filter MIPS

- Filter Type = MIPS
- Filter Label =
- MIPS Mode = Peptide
- Relax Restrictions when too few Precursors are Found = False

Filter IntensityThreshold

- Filter Type = IntensityThreshold
- Filter Label =
- Intensity Filter Type = IntensityThreshold
- Minimum Intensity = 5000

Filter ChargeState
Filter Type = ChargeState
Filter Label =
Include charge state(s) = 2-5
Include undetermined charge states = False

Filter DynamicExclusion
Filter Type = DynamicExclusion
Filter Label =
Share Dynamic exclusion list with other selected dynamic exclusion filters = False
Dynamic Exclusion Mode = Custom
Exclude after n times = 1
Exclusion duration (s) = 45
Excl. Mass Width = ppm
Mass tolerance high = 10
Mass tolerance low = 10
Exclude isotopes = True
Perform dependent scan on single charge state per precursor only = True

Filter Purity
Filter Type = Purity
Filter Label =
Purity Threshold (%) = 70
Purity Window = 0.7

Data Dependent Properties
Data Dependent Mode= Cycle Time

Scan Event 1

Scan ddMSnScan
Multiplex Ions Enabled = False
Isolation Window = Custom
Isolation Window (m/z) = 0.7
Isolation Offset = Off
Maximum number of multiplexed ions = 0
Reported Mass = Offset Mass
Collision Energy Mode = Fixed
Collision Energy Type = Normalized
HCD Collision Energy (%) = 36
Orbitrap Resolution = 45000
First Mass (m/z) = 110
Microscans = 1
AGC Target = Standard
Maximum Injection Time Mode = Auto
DataType = Centroid
Source Fragmentation = False
Scan Description =
