# Supplementary material for: Integrative Proteomic Analysis Implicates Inhibition of Intracellular Protein Trafficking in Therapy-Induced Migrastasis in Prostate Cancer
Source: Proteomes. 2026 Aug 28;14(3):45. doi: 10.3390/proteomes14030045 (PMC13611321; doi:10.3390/proteomes14030045)
Supplement: Supplementary file 1 [file proteomes-14-00045-s001.zip › Proteomics experiments Excel Sheets and LC_MS details_/UNMC Proteomics Core LC_MS parameters/MSmethod_RG.pdf]

Orbitrap Fusion Lumos Method Summary

Creator: VCRDRC1050LUMOS\Administrator Last Modified: 10/08/2021 12:02:50 by VCRDRC1050LUMOS\Administrator

Global Settings
- Use Static Source Gasses
- Use Ion Source Settings from Tune = Not Checked
- Method Duration (min)= 155
- Ion Source Type = NSI
- Spray Voltage = Static
- Spray Voltage: Positive Ion (V) = 1900
- Spray Voltage: Negative Ion (V) = 600
- Gas Mode = Static
- Infusion Mode (LC)= False
- Sweep Gas (Arb) = 0
- Ion Transfer Tube Temp (°C) = 275
- APPI Lamp = Not in use
- FAIMS Mode = Not Installed
- Internal Mass Calibration = EASY-IC
- Application Mode = Peptide
- Pressure Mode = Standard
- Default Charge State = 2
- Advanced Peak Determination = True

Experiment 1
- Experiment Name = Universal Method
- Start Time (min) = 0
- End Time (min) = 155
- Cycle Time (sec) = 3

  - Scan MasterScan
    - Desired minimum points across the peak = 6
    - MSn Level = 1
    - Use Wide Quad Isolation = True
    - Detector Type = Orbitrap
    - Orbitrap Resolution = 120K
    - Mass Range = Normal
    - Scan Range (m/z) = 375-1500
    - Maximum Injection Time (ms) = 50
    - AGC Target = 400000
    - Normalized AGC Target = 100%
    - Microscans = 1
    - Maximum Injection Time Type = Custom
    - RF Lens (%) = 30
    - Use ETD Internal Calibration = True
    - DataType = Profile
    - Polarity = Positive
    - Source Fragmentation = False
    - Scan Description =
    - Enhanced Resolution Mode = Off

  - Filter MIPS
    - Relax Restrictions when too few Precursors are Found = True
    - MIPS Mode = Peptide

  - Filter ChargeState

Include charge state(s) = 2-5
Include undetermined charge states = False

Filter DynamicExclusion
Exclude after n times = 1
Exclusion duration (s) = 60
Mass Tolerance = ppm
Mass tolerance low = 10
Mass tolerance high = 10
Use Common Settings = False
Exclude isotopes = True
Perform dependent scan on single charge state per precursor only = False

Filter IntensityThreshold
Maximum Intensity = 1E+20
Minimum Intensity = 25000
Relative Intensity Threshold = 0
Intensity Filter Type = IntensityThreshold

Data Dependent Properties
Data Dependent Mode= Cycle Time

Scan Event 1

Scan ddMSnScan
Desired minimum points across the peak = 6
MSn Level = 2
Isolation Mode = Quadrupole
Enable Intelligent Product Acquisition for MS2 Isolation = False
Isolation Window = 1.6
Isolation Offset = Off
Reported Mass = Original Mass
Multi-notch Isolation = False
Scan Range Mode = Auto
Scan Priority= 1
Collision Energy Mode = Fixed
ActivationType = HCD
Collision Energy (%) = 35
Detector Type = Orbitrap
Orbitrap Resolution = 15K
Maximum Injection Time (ms) = 22
AGC Target = 50000
Inject ions for all available parallelizable time = False
Normalized AGC Target = 100%
Microscans = 1
Maximum Injection Time Type = Dynamic
Use ETD Internal Calibration = False
DataType = Centroid
Polarity = Positive
Source Fragmentation = False
Scan Description =
Time Mode = Unscheduled
Enhanced Resolution Mode = Off
