# Supplementary material for: Integrative Proteomic Analysis Implicates Inhibition of Intracellular Protein Trafficking in Therapy-Induced Migrastasis in Prostate Cancer
Source: Proteomes. 2026 Aug 28;14(3):45. doi: 10.3390/proteomes14030045 (PMC13611321; doi:10.3390/proteomes14030045)
Supplement: Supplementary file 1 [file proteomes-14-00045-s001.zip › Supplementary Figures and Tables.pdf]

Supplementary Information (Figures and Tables) to the manuscript:

Integrative Proteomic Analysis Implicates Inhibition of Intracellular Protein Trafficking in Therapy-Induced Migrastasis in Prostate Cancer

Weining Chen [1,2,3,†], Saadyeh Rashidi [4,†], Henry C. -H. Law [2,3], Fangfang Qiao [1], Johnny W. Zigmond [2,3], Katelyn L. O'Neill [2,3], Nicholas T. Woods [2,3], Chittibabu Guda [4,*] and Raymond C. Bergan [1,*]

[1]Stony Brook Cancer Center, Stony Brook University, Stony Brook, NY 11794, USA;
[2]Eppley Institute for Research in Cancer, University of Nebraska Medical Center, Omaha, NE 68198, USA;
[3]Fred & Pamela Buffett Cancer Center, University of Nebraska Medical Center, Omaha, NE 68198, USA
[4]Department of Genetics, Cell Biology, and Anatomy, College of Medicine, University of Nebraska Medical Center, Omaha, NE 68198, USA
*Correspondence
[†]These authors contributed equally to this work.

Supplementary Table S1. Top 10 Compartment Enrichment Analysis of Proteins with Decreased Abundance Following KBU2046 Treatment.

Index	Name	P-value	Adjusted p-value	Odds Ratio	Combined score
1	TRANSPORT VESICLE MEMBRANE	0.0001305	0.02313	17.54	156.89
2	COATED VESICLE MEMBRANE	0.0002224	0.02313	15.19	127.79
3	COATED VESICLE	0.001089	0.07548	9.84	67.11
4	CLATHRIN VESICLE COAT	0.001497	0.07785	39.28	255.48
5	TRANSPORT VESICLE	0.002328	0.08193	7.94	48.17
6	CLATHRIN COAT	0.002363	0.08193	30.77	186.11
7	ER TO GOLGI TRANSPORT VESICLE MEMBRANE	0.004324	0.1285	22.31	121.45
8	VESICLE COAT	0.005332	0.1386	19.96	104.45
9	RECYCLING ENDOSOME MEMBRANE	0.007637	0.1531	16.48	80.31
10	MITOCHONDRIAL IRON-SULFUR CLUSTER ASSEMBLY COMPLEX	0.009217	0.1531	138.60	649.60

Supplementary Table S1. Gene Ontology compartment analysis using the COMPARTMENTS curated database for the protein fraction with decreased abundance identified in the global whole cell proteomic analysis of PC3 cells treated with 10 μM KBU2046. The table details the top 10 enriched cellular components. Metrics provided include p-value, adjusted p-value, odds ratio, and combined score for each identified compartment. The complete dataset is available in the Supplementary Materials (Bioinformatics Raw Data).

Supplementary Table S2. Top 10 Compartment Enrichment Analysis of Proteins with Increased Abundance Following KBU2046 Treatment.

Index	Name	P-value	Adjusted p-value	Odds Ratio	Combined score
1	MITOCHONDRIAL MEMBRANE	5.831e-7	0.0001361	7.25	104.04
2	MITOCHONDRIAL ENVELOPE	0.000001143	0.0001361	6.78	92.70
3	ORGANELLE ENVELOPE	0.000003735	0.0002963	5.18	64.73
4	ORGANELLE INNER MEMBRANE	0.00001593	0.0009477	7.00	77.28
5	MITOCHONDRIAL INNER MEMBRANE	0.00006663	0.003172	6.58	63.32
6	MITOCHONDRION	0.00009233	0.003662	4.26	39.59
7	U2 SNRNP	0.002825	0.08988	28.28	166.01
8	MITOCHONDRIAL OUTER MEMBRANE	0.003021	0.08988	7.20	41.78
9	SITE OF DOUBLE-STRAND BREAK	0.003604	0.09261	10.50	59.07
10	ORGANELLE OUTER MEMBRANE	0.004467	0.09261	6.43	34.80

Supplementary Table S2. Gene Ontology compartment analysis using the COMPARTMENTS curated database for the protein fraction with increased abundance identified in the global whole cell

proteomic analysis of PC3 cells treated with 10 μM KBU2046. The table details the top 10 enriched cellular components. Metrics provided include p-value, adjusted p-value, odds ratio, and combined score for each identified compartment. The complete dataset is available in the Supplementary Materials (Bioinformatics Raw Data).

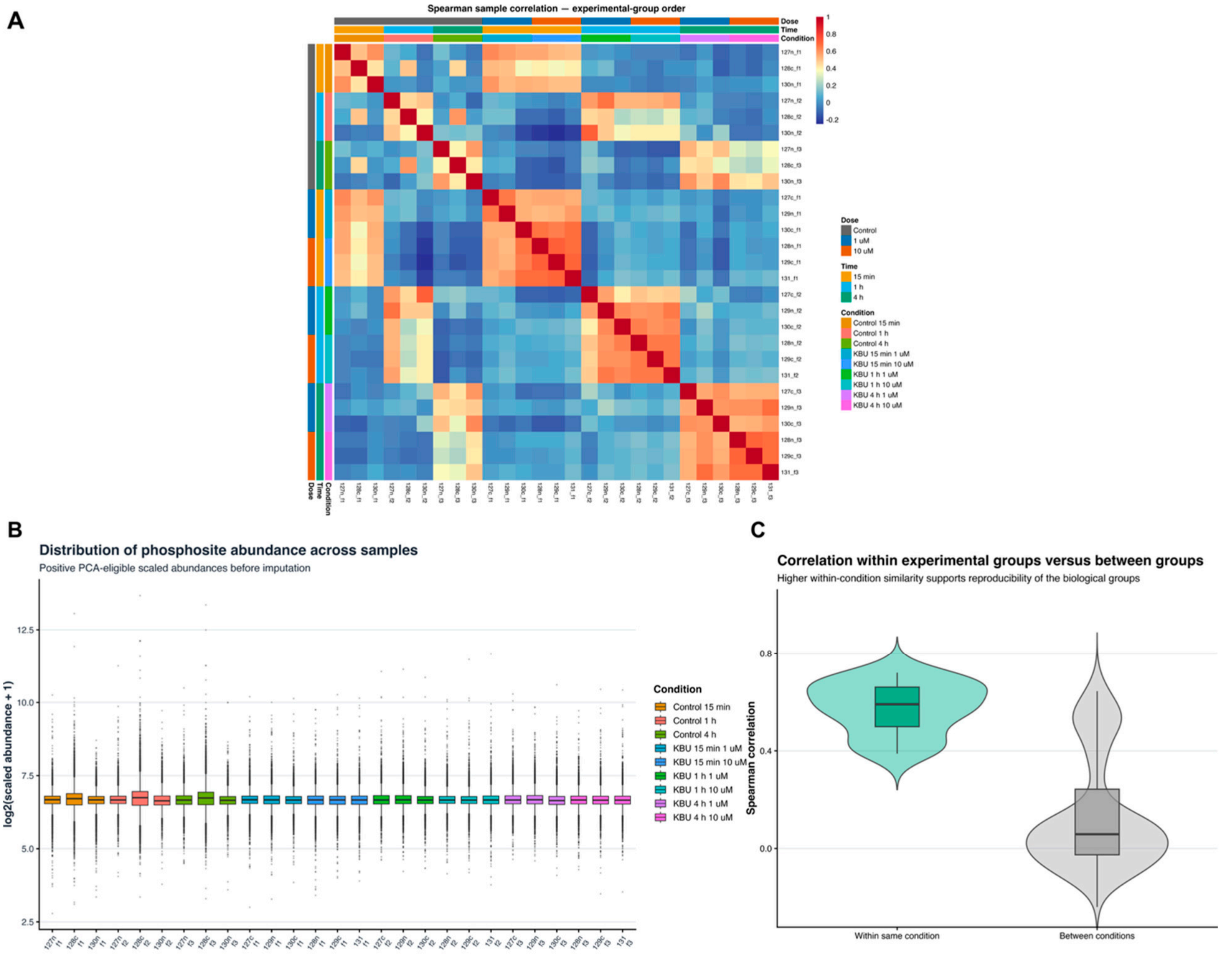

Supplementary Figure S1. Sample-level phosphoproteomic quality control and correlation structure. (**A**) Spearman correlation heatmap of 16,054 high-confidence phosphosite-level features, with samples arranged by experimental condition. (**B**) Distribution of positive scaled phosphosite abundances before imputation. (**C**) Comparison of Spearman correlations for sample pairs from the same experimental condition and pairs from different conditions. Condition-matched samples showed substantially greater similarity than unmatched samples.

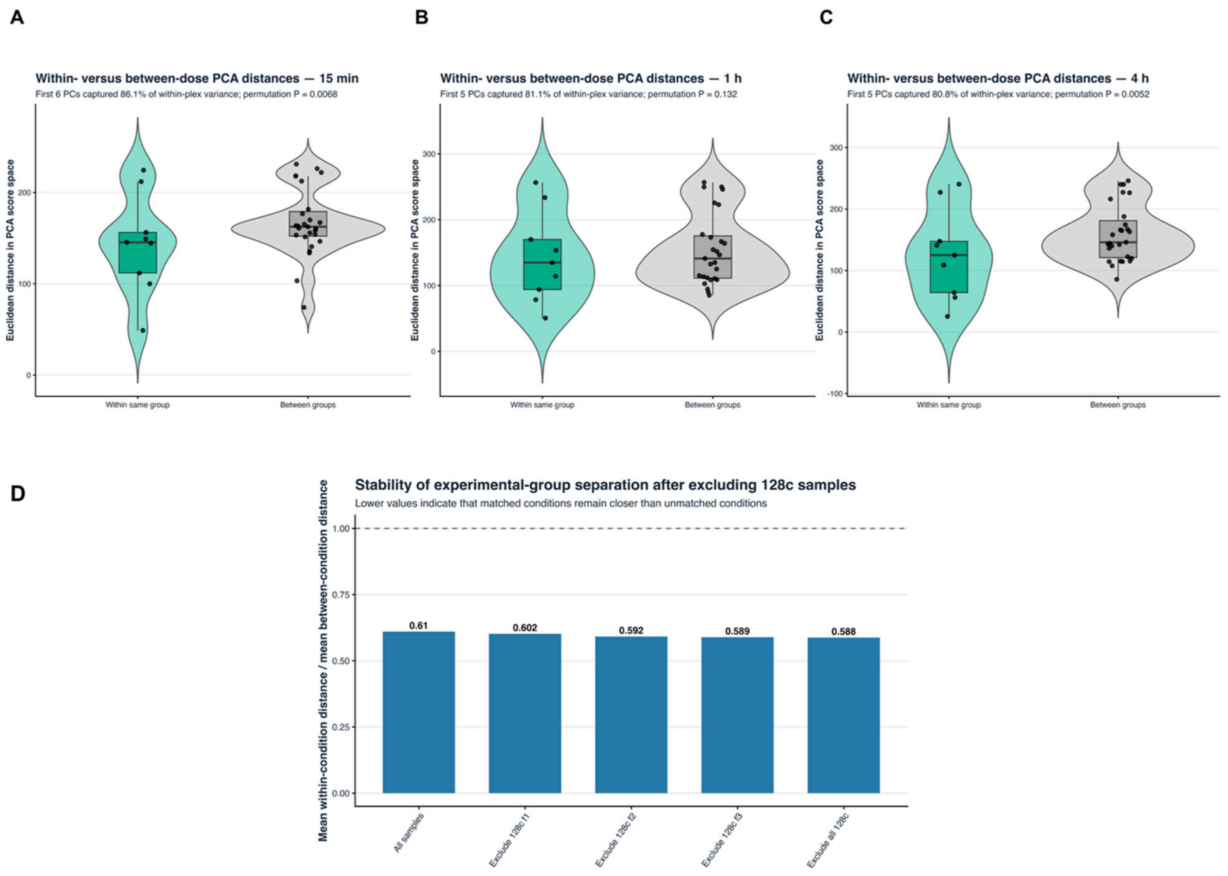

Supplementary Figure S2. Plex-aware PCA-distance and reporter-channel sensitivity analyses. (**A–C**) Comparison of within-dose and between-dose distances in PCA score space for the 15-minute, 1-hour, and 4-hour TMT plexes, respectively. PCA distances were calculated using the first components explaining at least 80% of within-plex variance. Dose-label permutation tests supported dose-associated separation at 15 minutes and 4 hours, whereas the 1-hour comparison did not reach statistical significance. (**D**) Stability of experimental-group separation after excluding each 128C sample individually or all three 128C samples. Ratios below one indicate that condition-matched samples remained closer than unmatched samples.

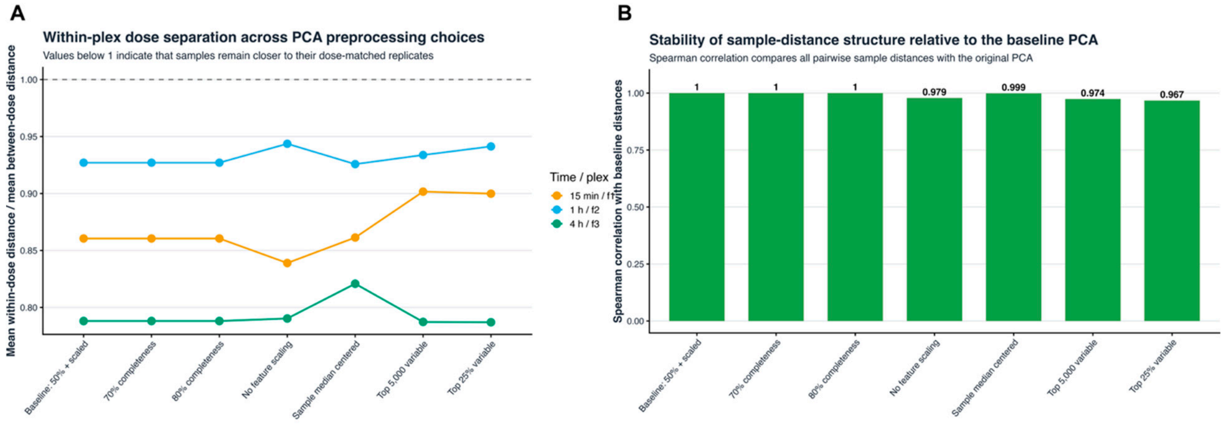

Supplementary Figure S3. Robustness of PCA sample relationships to preprocessing choices. (**A**) Within-plex dose-separation ratios following alternative completeness thresholds, feature scaling, sample-median centering, and variable-feature selection. Ratios below one indicate that samples were closer to dose-matched replicates than to other dose groups. (**B**) Spearman correlations between pairwise sample distances from each alternative PCA and the baseline PCA. Correlations ranged from 0.967 to 1.000, demonstrating preservation of the overall sample-distance structure.

Supplementary Table S3. Summary of phosphoproteomic sample-level quality control and PCA robustness analyses.

QC_category	Metric	Result	Interpretation
**Dataset**	Samples evaluated	27	All samples were retained for QC and sensitivity analyses.
**Dataset**	PCA-eligible phosphosite features	16,054	High-confidence phosphosite-level features were evaluated consistently across QC analyses.
**Sample-level abundance**	Maximum unavailable or non-positive features	0%	No sample showed reduced quantitative coverage among retained features.
**Correlation**	Median within-condition Spearman correlation	0.591	Replicates from the same experimental condition showed coherent phosphosite patterns.
**Correlation**	Median between-condition Spearman correlation	0.058	Within-condition similarity was substantially greater than between-condition similarity.
**Within-plex dose separation**	15-minute permutation test	P = 0.0068	Dose-associated separation was supported at 15 minutes.
**Within-plex dose separation**	1-hour permutation test	P = 0.132	The directional dose pattern was present but not statistically significant at 1 hour.
**Within-plex dose separation**	4-hour permutation test	P = 0.0052	Dose-associated separation was supported at 4 hours.
**128C sensitivity**	Group-distance ratio with all samples	0.610	Condition-matched samples were closer than unmatched samples.
**128C sensitivity**	Group-distance ratio after excluding all 128C samples	0.588	Removing all 128C samples produced only a modest change and did not eliminate experimental structure.
**Preprocessing robustness**	Range of within-condition/between-condition distance ratios	0.536–0.610	Condition-matched samples remained closer under every preprocessing strategy.
**Preprocessing robustness**	Spearman stability of pairwise sample distances	0.967–1.000	The overall sample-distance structure was almost unchanged across preprocessing choices.

Supplementary Table S3. Summary of phosphoproteomic sample-level quality control and PCA robustness analyses. The table reports sample-level, correlation, within-plex, reporter-channel, and PCA-preprocessing robustness metrics.

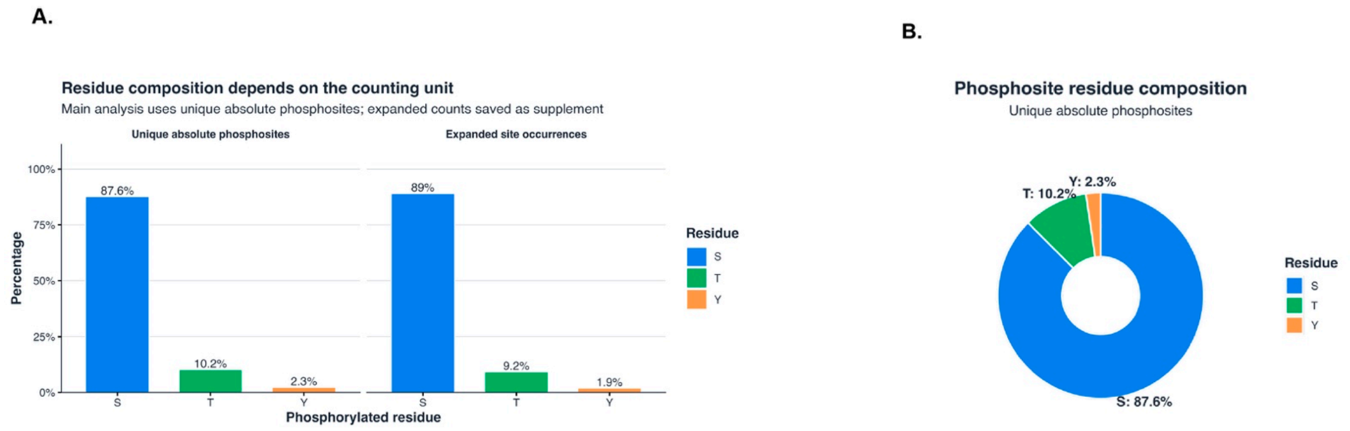

Supplementary Figure S4. Phosphosite residue composition sensitivity and distribution. (**A**) Bar charts comparing the residue composition of phosphorylated residues calculated via unique absolute phosphosites versus expanded site occurrences. (**B**) A pie chart visualizing the proportional residue composition of unique absolute phosphosites, demonstrating a distribution of 87.6% serine, 10.2% threonine, and 2.3% tyrosine. The canonical mammalian residue distribution is robustly maintained irrespective of the bioinformatic counting methodology.

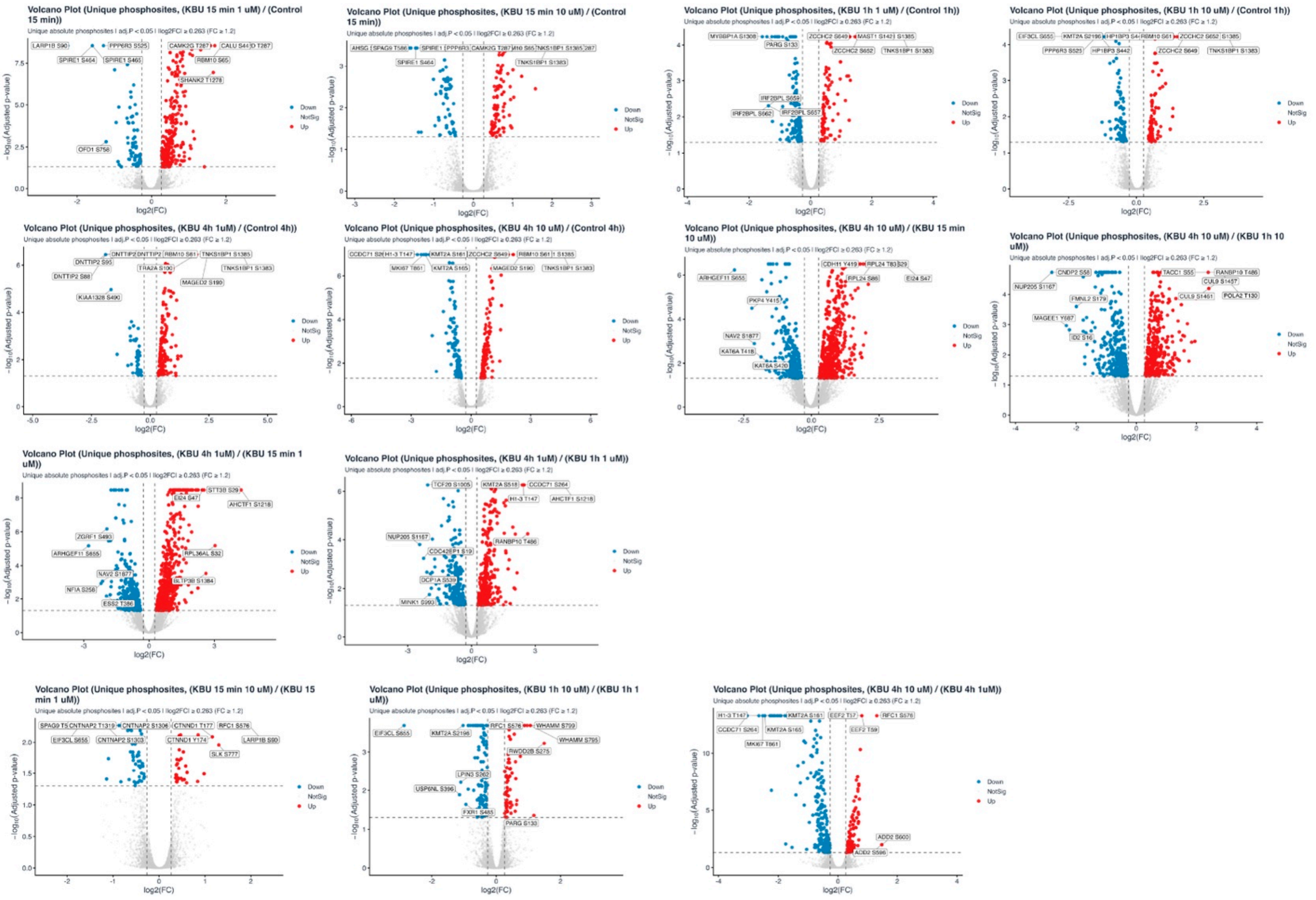

Supplementary Figure S5. Standard Threshold Volcano Plot of Phosphosites. Volcano plot highlighting the global distribution of unique absolute phosphosites with increased and decreased abundance across KBU2046 treatment conditions and time points. Significant hits are defined by the standard threshold of |Log2 FC| ≥ 0.26 and adjusted p-value < 0.05. Red points indicate phosphosites with increased abundance, blue points indicate phosphosites with decreased abundance, and grey points indicate phosphosites without significant alteration. Individual panels show dose- and time-specific comparisons, including KBU2046-treated samples versus matched controls and selected dose-to-dose comparisons.

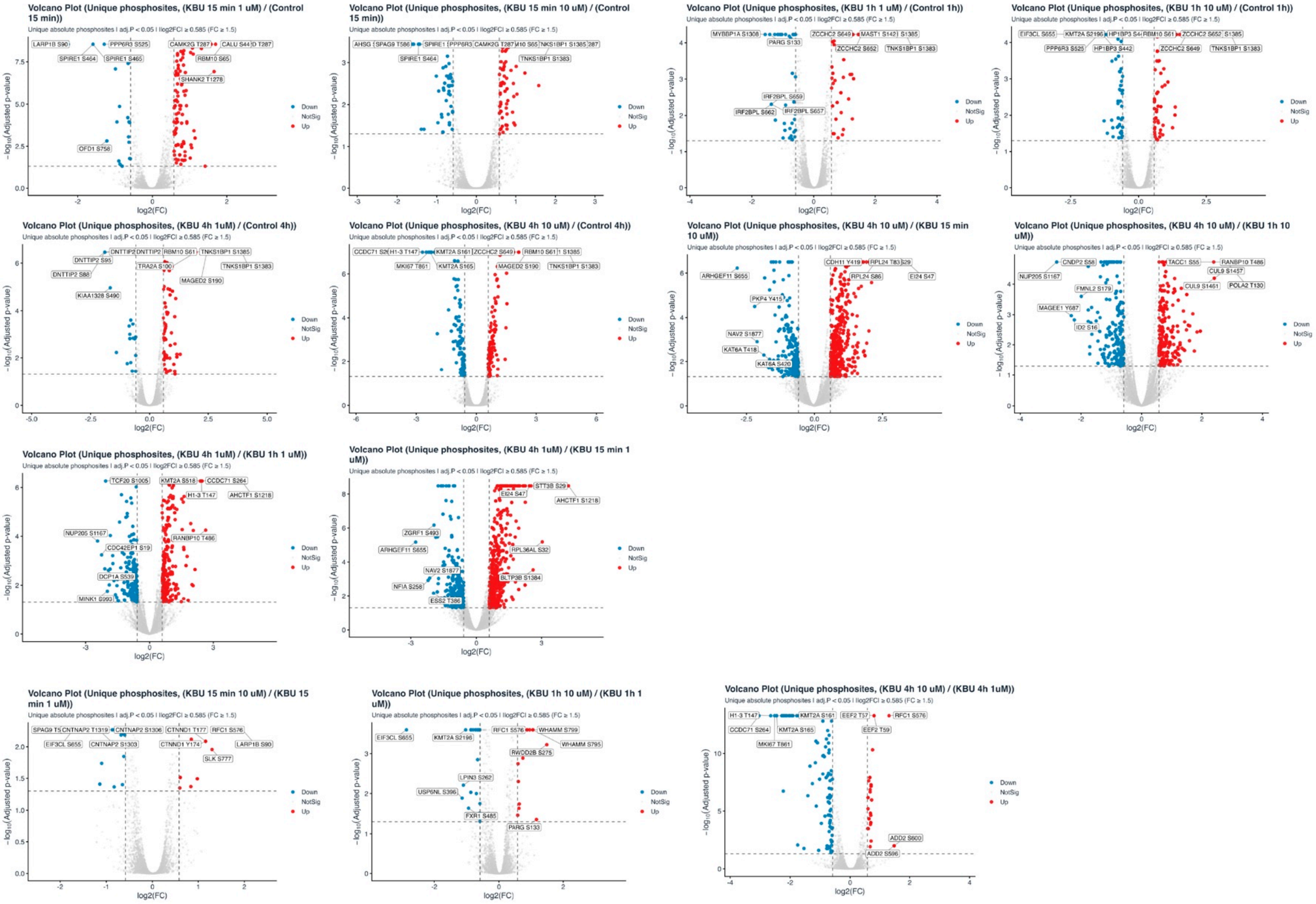

Supplementary Figure S6. High-Stringency Threshold Volcano Plot of Phosphosites. Volcano plot highlighting the global distribution of unique absolute phosphosites with increased and decreased abundance across KBU2046 treatment conditions and time points under stringent statistical filtering. Significant hits are restricted to a high-stringency threshold of |Log2 FC| ≥ 0.58 and adjusted p-value < 0.05. Red points indicate phosphosites with increased abundance, blue points indicate phosphosites with decreased abundance, and grey points indicate phosphosites without significant alteration.

Supplementary Table S4. Quantitative profile of individual MAPK14 phosphorylation entries corresponding to Figure 9B.

Phosphorylation Site	KBU2046 Dose	Log2 Fold Change	Adjusted p-value	Meets Strict Threshold
Site 1: 2xPhospho T180 and Y182	1 µM	1.02	7.11×10^{-10}	Yes
Site 1: 2xPhospho T180 and Y182	10 µM	1.11	3.75×10^{-6}	Yes
Site 2: 2xPhospho T180 and Y182	1 µM	0.63	9.74×10^{-6}	Yes
Site 2: 2xPhospho T180 and Y182	10 µM	0.55	0.0489	No
Site 3: 1xPhospho Y182	1 µM	0.04	0.977	No
Site 3: 1xPhospho Y182	10 µM	−0.10	0.988	No

Supplementary Table S4. Quantitative profile of individual MAPK14 phosphorylation entries corresponding to Figure 9B. This table includes individual MAPK14 peptide phosphorylation entries detected at 4 hours post-treatment with 1 µM or 10 µM KBU2046, compared with the vehicle control. The columns detail the specific phosphorylation site, KBU2046 dose, Log2 FC, adjusted p-value, and whether the entry meets the strict significance threshold ($|\text{Log2 FC}| \geq 0.58$ and adjusted p-value < 0.05).

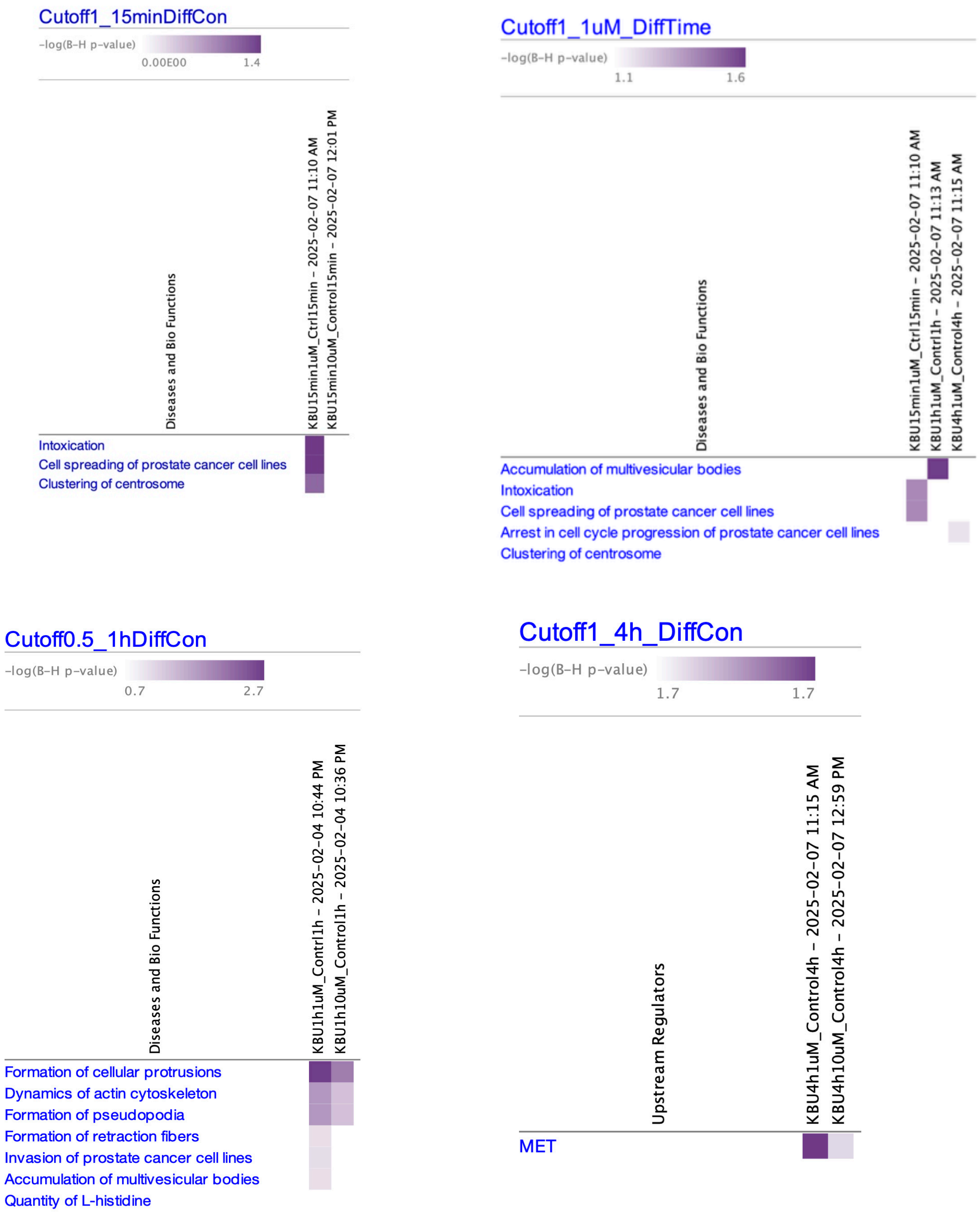

Supplementary Figure S7. Expanded pathway enrichment heatmaps from temporal phosphoproteomic analysis. High-resolution uncropped visualizations of the Ingenuity Pathway Analysis Disease and Bio Functions mapping across the evaluated treatment concentrations and time intervals of Figures 8A, 8B, 8C and 9A.

Protein FDR Confidence: Combined	Accession	Description	Sum PEP Score	Coverage [%]	# Peptides	# PSMs	# Unique Peptides	# Razor Peptides	Abundance Ratio (log2): (KBU 15 min 1 uM) / (Control 15 min)	Abundance Ratio (log2): (KBU 15 min 10 uM) / (Control 15 min)	Abundance Ratio (log2): (KBU 1h 1 uM) / (Control 1h)	Abundance Ratio (log2): (KBU 1h 10 uM) / (Control 1h)	Abundance Ratio (log2): (KBU 4h 10 uM) / (Control 4h)	Abundance Ratio (log2): (KBU 4h 1uM) / (Control 4h)	Abundance Ratio (log2): (KBU 15 min 10 uM) / (KBU 15 min 1 uM)	Abundance Ratio (log2): (KBU 4h 1uM) / (KBU 15 min 1 uM)	Abundance Ratio (log2): (KBU 4h 10 uM) / (KBU 15 min 10 uM)	Abundance Ratio (log2): (KBU 1h 10 uM) / (KBU 1h 1 uM)	Abundance Ratio (log2): (KBU 4h 1uM) / (KBU 1h 1 uM)	Abundance Ratio (log2): (KBU 4h 10 uM) / (KBU 1h 10 uM)	Abundance Ratio (log2): (KBU 4h 10 uM) / (KBU 4h 1uM)	Abundance Ratio Adj. P-Value: (KBU 15 min 1 uM) / (Control 15 min)	Abundance Ratio Adj. P-Value: (KBU 15 min 10 uM) / (Control 15 min)	Abundance Ratio Adj. P-Value: (KBU 1h 1 uM) / (Control 1h)	Abundance Ratio Adj. P-Value: (KBU 1h 10 uM) / (Control 1h)	Abundance Ratio Adj. P-Value: (KBU 4h 10 uM) / (Control 4h)	Abundance Ratio Adj. P-Value: (KBU 4h 1uM) / (Control 4h)	Abundance Ratio Adj. P-Value: (KBU 15 min 10 uM) / (KBU 15 min 1 uM)	Abundance Ratio Adj. P-Value: (KBU 4h 1uM) / (KBU 15 min 1 uM)	Abundance Ratio Adj. P-Value: (KBU 4h 10 uM) / (KBU 15 min 10 uM)	Abundance Ratio Adj. P-Value: (KBU 1h 10 uM) / (KBU 1h 1 uM)	Abundance Ratio Adj. P-Value: (KBU 4h 1uM) / (KBU 1h 1 uM)	Abundance Ratio Adj. P-Value: (KBU 4h 10 uM) / (KBU 1h 10 uM)	Abundance Ratio Adj. P-Value: (KBU 4h 10 uM) / (KBU 4h 1uM)
High	Q9UIG0	Tyrosine-protein kinase BAZ1B OS=Homo sapiens OX=9606 GN=BAZ1B PE=1 SV=2	54.68	6	8	110	8	0	0.02	0.15	0.31	0.22	-1.09	0.17	0.13	1.3	-0.09	-0.08	1.04	-0.14	-1.26	0.97421639	0.81191958	0.00010969	0.25101848	3.5731E-15	0.39804462	0.61667383	7.2068E-17	0.78139268	0.33242921	8.27E-17	0.50069601	9.883E-16
	Confidence	Annotated Sequence	Modifications	Positions in Master Proteins	Modifications in Master Proteins	PEP	# PSMs	# Missed Cleavages	Abundance Ratio (log2): (KBU 15 min 1 uM) / (Control 15 min)	Abundance Ratio (log2): (KBU 15 min 10 uM) / (Control 15 min)	Abundance Ratio (log2): (KBU 1h 1 uM) / (Control 1h)	Abundance Ratio (log2): (KBU 1h 10 uM) / (Control 1h)	Abundance Ratio (log2): (KBU 4h 10 uM) / (Control 4h)	Abundance Ratio (log2): (KBU 4h 1uM) / (Control 4h)	Abundance Ratio (log2): (KBU 15 min 10 uM) / (KBU 15 min 1 uM)	Abundance Ratio (log2): (KBU 4h 1uM) / (KBU 15 min 1 uM)	Abundance Ratio (log2): (KBU 4h 10 uM) / (KBU 15 min 10 uM)	Abundance Ratio (log2): (KBU 1h 10 uM) / (KBU 1h 1 uM)	Abundance Ratio (log2): (KBU 4h 1uM) / (KBU 1h 1 uM)	Abundance Ratio (log2): (KBU 4h 10 uM) / (KBU 1h 10 uM)	Abundance Ratio (log2): (KBU 4h 10 uM) / (KBU 4h 1uM)	Abundance Ratio Adj. P-Value: (KBU 15 min 1 uM) / (Control 15 min)	Abundance Ratio Adj. P-Value: (KBU 15 min 10 uM) / (Control 15 min)	Abundance Ratio Adj. P-Value: (KBU 1h 1 uM) / (Control 1h)	Abundance Ratio Adj. P-Value: (KBU 1h 10 uM) / (Control 1h)	Abundance Ratio Adj. P-Value: (KBU 4h 10 uM) / (Control 4h)	Abundance Ratio Adj. P-Value: (KBU 4h 1uM) / (Control 4h)	Abundance Ratio Adj. P-Value: (KBU 15 min 10 uM) / (KBU 15 min 1 uM)	Abundance Ratio Adj. P-Value: (KBU 4h 1uM) / (KBU 15 min 1 uM)	Abundance Ratio Adj. P-Value: (KBU 4h 10 uM) / (KBU 15 min 10 uM)	Abundance Ratio Adj. P-Value: (KBU 1h 10 uM) / (KBU 1h 1 uM)	Abundance Ratio Adj. P-Value: (KBU 4h 1uM) / (KBU 1h 1 uM)	Abundance Ratio Adj. P-Value: (KBU 4h 10 uM) / (KBU 1h 10 uM)	Abundance Ratio Adj. P-Value: (KBU 4h 10 uM) / (KBU 4h 1uM)
	High	[K].KSDGACDSPSSDK.[E]	2xTMT6plex [K1; K13]; 1xTMT6plex [N-Term]; 1xCarbamidomethyl [C6]; 1xPhospho [S]	Q9UIG0 [151-163]		6.448E-06	8	1	-0.02	0.01	0.15	0.19	-1.03	-0.09	0.03	0.97	0	0.04	0.78	-0.2	-0.94	0.9999935	0.99970258	0.88060296	0.88819896	4.0147E-07	0.94573975	0.9951557	1.584E-05	0.99249786	0.99277677	0.00036636	0.63455862	2.6534E-15
	High	[R].RQSLELQK.[C]	1xTMT6plex [N-Term]; 1xTMT6plex [K8]; 1xPhospho [S3(100)]	Q9UIG0 [1340-1347]	Q9UIG0 1xPhospho [S1342(100)]	0.0157706	8	1	0.04	0.19	0.32	0.13	-1.08	0.07	0.14	1.07	-0.21	-0.19	0.81	-0.14	-1.14	0.99127825	0.91657579	0.22160818	0.97199879	9.0143E-09	0.94829975	0.91580018	5.683E-09	0.60008412	0.57816822	4.4892E-05	0.81083994	2.6534E-15
	High	[K].SLSGSPLK.[V]	1xTMT6plex [N-Term]; 1xTMT6plex [K8]; 1xPhospho [S5(100)]	Q9UIG0 [345-352]	Q9UIG0 1xPhospho [S349(100)]	0.00333391	10	0	-0.18	0.14	0.15	0.24	-1.21	0.26	0.32	1.63	-0.15	0.09	1.65	0.09	-1.47	0.76708004	0.96635669	0.88419638	0.81330658	3.8255E-11	0.26788757	0.26858809	3.3305E-17	0.73125532	0.96417052	3.699E-17	0.87348378	2.6534E-15
	High	[K].DHTVSGDEDYCPR.[S]	1xTMT6plex [N-Term]; 1xCarbamidomethyl [C11]; 1xPhospho [S5(100)]	Q9UIG0 [943-955]	Q9UIG0 1xPhospho [S947(100)]	1.7595E-05	12	0	0.31	0.24	0.32	0.23	-1.39	-0.06	-0.07	0.29	-0.97	-0.09	0.21	-1.04	-1.34	0.7106658	0.93442645	0.58207061	0.88062595	4.3261E-05	0.98581562	0.99967007	0.68487618	0.03103689	0.99277677	0.77888921	0.01845778	1.2251E-09
	High	[K].KSDGACDSPSSDK.[E]	2xTMT6plex [K1; K13]; 1xTMT6plex [N-Term]; 1xCarbamidomethyl [C6]; 2xPhospho [S8(100); S]	Q9UIG0 [151-163]	Q9UIG0 2xPhospho [S158(100); S]	1.4287E-05	10	1	-0.12	0.13	0.25	0.15	-1.19	-0.24	0.25	0.94	-0.27	-0.1	0.77	-0.08	-0.96	0.90795397	0.97256667	0.37938931	0.97225438	4.8242E-10	0.51886607	0.47574076	1.2196E-05	0.37955689	0.89306958	0.00016934	0.8841294	2.6534E-15
	High	[K].SLSGSPLK.[V]	1xTMT6plex [N-Term]; 1xTMT6plex [K8]; 2xPhospho [S3(100); S5(100)]	Q9UIG0 [345-352]	Q9UIG0 2xPhospho [S347(100); S349(100)]	0.133674	4	0	0.04	-0.06	0.45	0.13	-0.96	0.34	-0.1	0.97	-0.24	-0.31	0.48	-0.51	-1.3	0.99684316	0.99970258	0.0094127	0.96796977	3.4415E-06	0.05829184	0.97807273	5.4565E-07	0.57795557	0.04368274	0.014888	0.05243093	2.6534E-15
	High	[R].SDVQEESEGSDTDDNK.[D]	1xTMT6plex [N-Term]; 1xTMT6plex [K16]; 2xPhospho [S7(100); S10(100)]	Q9UIG0 [699-714]	Q9UIG0 2xPhospho [S705(100); S708(100)]	2.5244E-09	30	0	0.15	0.24	0.33	0.27	-0.97	0.16	0.09	1.31	0.1	-0.07	1.06	-0.01	-1.13	0.71681731	0.77375392	0.04497891	0.64011405	2.2487E-08	0.72329905	0.9795745	3.3305E-17	0.71372988	0.89643482	3.699E-17	0.98444842	2.6534E-15
	High	[R].RSDVQEESEGSDTDDNK.[D]	1xTMT6plex [N-Term]; 1xTMT6plex [K17]; 2xPhospho [S8(99.6); S11(94.3)]	Q9UIG0 [698-714]	Q9UIG0 2xPhospho [S705(99.6); S708(94.3)]	3.5501E-07	16	1	-0.06	0.17	0.25	0.28	-1.38	0.19	0.22	1.57	-0.23	0.02	1.29	-0.3	-1.57	0.9836143	0.93547971	0.3589182	0.63774409	6.4867E-15	0.58342244	0.53233803	4.4344E-15	0.53425801	0.99921831	3.3286E-11	0.27918876	2.6534E-15

Supplementary Figure S8. Quantitative evaluation of BAZ1B protein abundance and site-specific phosphorylation status. The data summary contains mass spectrometry quantification for the BAZ1B protein and associated phosphopeptides across evaluated time intervals and treatment concentrations. Values include normalized abundance ratios, adjusted p-values, and specific phosphorylated residues. Detailed data sheets are provided in the Supplementary Data.
